# Supplementary material for: Route to diagnosis of degenerative cervical myelopathy in a UK healthcare system: a retrospective cohort study
Source: BMJ Open. 2019 May 5;9(5):e027000. doi: 10.1136/bmjopen-2018-027000 (PMC6501948; doi:10.1136/bmjopen-2018-027000)
Supplement: Author's manuscript [file bmjopen-2018-027000.draft_revisions.pdf]

BMJ Open is committed to open peer review. As part of this commitment we make the peer review history of every article we publish publicly available.

When an article is published we post the peer reviewers' comments and the authors' responses online. We also post the versions of the paper that were used during peer review. These are the versions that the peer review comments apply to.

The versions of the paper that follow are the versions that were submitted during the peer review process. They are not the versions of record or the final published versions. They should not be cited or distributed as the published version of this manuscript.

BMJ Open is an open access journal and the full, final, typeset and author-corrected version of record of the manuscript is available on our site with no access controls, subscription charges or pay-per-view fees (http://bmjopen.bmj.com).

If you have any questions on BMJ Open's open peer review process please email info.bmjopen@bmj.com

BMJ Open

Delayed diagnosis in Degenerative Cervical Myelopathy: an overview of patient flow in a UK healthcare system

Journal:	*BMJ Open*
Manuscript ID	bmjopen-2018-027000
Article Type:	Research
Date Submitted by the Author:	09-Oct-2018
Complete List of Authors:	Hilton, Bryn; University of Cambridge<br>Tempest-Mitchell, Jennifer; University of Cambridge<br>Davies, Benjamin; University of Cambridge, Department of Clinical Neurosurgery<br>Kotter, Mark; University of Cambridge, Department of Clinical Neurosurgery; Ann McLaren Laboratory of Regenerative Medicine, Department of Clinical Neurosciences
Keywords:	Spine < ORTHOPAEDIC & TRAUMA SURGERY, Neurological injury < NEUROLOGY, NEUROLOGY

SCHOLARONE™
Manuscripts

Delayed diagnosis in Degenerative Cervical Myelopathy: an overview of patient flow in a UK healthcare system

Bryn Hilton[1], Jennifer Tempest-Mitchell[1], Benjamin M. Davies M.R.C.S B.Sc[2], Mark R.N. Kotter M.D. M.Phil. Ph.D. [2-3]

1) School of Clinical Medicine, University of Cambridge, Cambridge, UK
2) Academic Neurosurgery Unit, Department of Clinical Neurosurgery, University of Cambridge, Cambridge, UK
3) WT MRC Cambridge Stem Cell Institute, Anne McLaren Laboratory, University of Cambridge, UK

For peer review only

Key words:
Cervical, Myelopathy, Spondylosis, Degeneration

Word Count: *3008*

Reference Count: *32*

Acknowledgements:
MK is supported by NIHR Clinician Scientist Award (CS-2015-15-023)

Contributorship Statement

BH – study design, data collection, data analysis, manuscript writing.

JTM – data collection, manuscript editing.

BD – study design, manuscript editing.

MK – study design, manuscript editing.

Ethics Declarations
All data was collected as part of a service evaluation and appropriate prior permission was sought and granted.

Data Sharing Statement
No additional data available

Correspondence:
Dr. Mark R.N. Kotter

Department of Clinical Neurosciences

Ann McLaren Laboratory of Regenerative Medicine
University of Cambridge
West Forvie Building
Forvie Site Box 213
Hills Road
Cambridge
CB2 0SZ
UK

Email: mrk25@cam.ac.uk
Work telephone: 01223 747476
Work fax: 01223 763350

For peer review only

Objectives

Degenerative Cervical Myelopathy (DCM) presents insidiously, making initial diagnosis challenging. Surgery has been shown to prevent further disability but existing spinal cord damage may be permanent. Delays in surgery lead to increased disability and reduced post-operative improvements. Therefore, rapid surgical assessment is key to improving patient outcomes. Unfortunately, diagnosis of DCM in primary care is often delayed. This study aimed to characterise DCM patients' pathway to diagnosis and surgical assessment.

Methods

One year of cervical MRI scans conducted at a tertiary neurosciences centre (N=1123) were screened for cervical cord compression and a corresponding clinical diagnosis of myelopathy (N=43). Disease severity was approximated using a prospectively validated method for inferring modified Japanese Orthopaedic Association (i-mJOA) functional scoring from clinical documentation.

Results

Patients received a referral to secondary care 6.4±7.7 months after symptom onset. Cervical MRI scanning and neurosurgical review occurred 12.5±13.0 and 15.8±13.5 months after symptom onset respectively. i-mJOA was 16.0±1.7 at primary care assessment and 14.8±2.5 at surgical assessment. 61.0% of patients were offered operations. For those who received surgery, time between onset and surgery was 22.1±13.2 months.

Conclusions

Route to surgical assessment was heterogeneous and lengthy. Some patients deteriorated during this period. This study highlights the need for a streamlined pathway by which patients with cervical cord compression can receive timely assessment and treatment by a specialist. This would improve outcomes for patients using existing treatments.

Strengths and Limitations of this Study

·This is the largest study to characterise DCM patient flow and the first to do so within the UK healthcare system.

·This study clearly documents multi-factorial delays in diagnosis and management that are known to impact upon DCM patient outcomes. This study therefore provides the basis for logistical interventions that could improve patient outcomes using existing treatment strategies.

·Single-centre study. However, the centre covers a diverse population and given the dearth of research into diagnostic delays in DCM, this article provides a significant contribution.

For peer review only

·i-mJOA was developed to retrospectively allow characterisation of disease severity from patient case notes. Prospective and blinded validation confirmed its suitability as a proxy for the mJOA, the most common measure of disease severity.

For peer review only

Introduction

Degenerative Cervical Myelopathy (DCM) is an umbrella term for compression of the cervical spinal cord due to degenerative pathology, such as osteophyte formation, intervertebral disc protrusion, and ligament hypertrophy or ossification[1]. The estimated minimum incidence and prevalence of DCM's are 41 and 605 per 1,000,000 respectively in North America[1]. However, this is likely to be an underestimate as DCM often goes undiagnosed and epidemiology frequently relies on operative incidence.[1] In a recent study of 183 randomly selected adults aged 40-80, 59% had radiological evidence of cervical cord compression and 1% as yet undiagnosed DCM. The evolution of incidental cord compression is unclear, but in the only study of its kind Bednarik et al demonstrated 23% went onto develop DCM in a series of 199 patients.[2] These findings would suggest DCM is far more common than currently demonstrated.

DCM causes progressive neurological dysfunction leading to significant disability and reduction in quality of life for patients.[3] Symptoms include limb spasticity, numb and clumsy hands, sphincter dysfunction, neck and limb pain, imbalance, and limb weakness leading to falls.[4] These symptoms often arise and progress insidiously in either a step-wise or continuous manner.[5] The rate of progression varies between individuals and cannot currently be predicted.

A diagnosis of DCM requires congruent symptoms, clinical and MRI findings, typically evidence of spinal cord compression.[6] Surgery is the only evidence based treatment for DCM and has been shown to halt disease progression and

For peer review only

afford some improvement across a number of domains.[5,7,8] However few patients make a complete recovery. This is due to the limited regenerative capacity of the spinal cord; as a result existing damage is often permanent and leads to life-long disabilities. A recent study has demonstrated that DCM patients have amongst the lowest SF-36 scores of chronic diseases.[9] Therefore improving functional recovery in DCM is a major unmet clinical need.

Recent international guidelines advise all DCM patients should be assessed by a specialist service and surgery offered for moderate to severe disease, as well as any progressive disease.[10]

Prognostic factors for post-operative functional improvement have been extensively studied. The main predictor of outcome is pre-surgical morbidity. Detecting progressive disease and early intervention is therefore important. Moreover, longer pre-operative symptom duration is associated with poorer post-operative improvement.[11-16] In the largest cohort study of DCM patients, surgery within 6 months of symptom onset offered the best chance of recovery. In this context, rapid referral to a spinal surgeon followed by prompt surgical intervention when appropriate is key to minimising disability.

Unfortunately, DCM patients report significant delays in diagnosis.[17] This is corroborated in the study of Behrbalk et al (2013) who found that most patients in Israel wait more than two years for a diagnosis.[18] If this could be overcome, outcomes would improve based on existing treatment strategies. Whilst the delay in diagnosis is becoming better recognised, where and why the delays

For peer review only

occur remain unclear.

Healthcare system research aims to characterise patient pathways, to identify the 'where' and 'why' of delays, to allow targeted logistical interventions. This process has been successful in cancer presentation and management,[19-22] informing clinician- and public policy-based initiatives to improve cancer care.

Our objectives, therefore, were to characterize the DCM patient pathways within the UK healthcare system in order to support targeted interventions aimed at accelerating diagnosis and management. This is the first study of its kind in the field of DCM.

Methods

Study description

Restrospective, observational cohort study

Cohort Description

The radiological report of all cervical spine MRI scans conducted over one year in a single, tertiary neurosciences centre were screened for descriptions of cervical spinal cord compression. The centre serves an estimated population of 5.9 million, although a considerable proportion of spinal patients are initially seen and are imaged, and may be managed in secondary care centres.[22] The clinical records corresponding to these scans were examined and cross-matched against inclusion criteria: non-traumatic cord compression due to spinal degeneration, a clinical diagnosis of DCM and adequate clinical documentation to characterise

For peer review only

the referral pathway. Cases that met these criteria formed the cohort for this study (Figure 1).

Peripheral hospitals in our region use different electronic and paper record keeping systems from one another and from this tertiary centre. Therefore, this study only included patients imaged in our centre, as this permitted full access to clinical documentation required to accurately document patient pathways.

Patient and Public Involvement

This study was developed in response to patient reports of frequent and long delays in their diagnosis and management. These reports came from the community of myelopathy.org, an international DCM charity and were reconfirmed as a priority research area during a Patient and Public Involvement day at the University of Cambridge. Some of these interviews were featured in a video documentary for Cambridge TV. While patients drove the theme for the study through their testimonials, they were not involved in the exact design of the study, which was informed by local referral pathways and literature on DCM. Once published, this study will be shared through myelopathy.org to make its findings widely available to DCM patients and carers.

Data Collection

All relevant clinical records (assessments, correspondences, inpatient documentation) were examined for the cohort cases. Demographic data was collected: age at time of onset, sex, relevant commodities, previous episodes of DCM and previous cervical surgery.

For peer review only

Retrospective Assessment of Disease Severity

The mJOA is a common and well-validated assessment of disability in DCM.[10,23] A retrospective method for assessment of the patient's modified Japanese Orthopaedic Association [mJOA] score was developed (Table 1) and prospectively validated (N=13) using a Kappa statistic. This was termed the inferred mJOA [i-mJOA]. imJOA was developed as a research tool for this study to allow retrospective assessment of change in DCM disability, as grading tools are not routinely employed in clinical consultations. It was not specifically developed for wider usage, which would require thorough external validation prior to application.

For peer review only

Table 1.

	Score	mJOA	imJOA
**Upper Limb Motor Dysfunction**	0	Inability to move hands	Power 0/5 or 1/5<br>No movement
	1	Inability to eat with a spoon but able to move hands	Power 2/5<br>Significant weakness/stiffness/clumsiness/impairment to daily living.<br>Significant assistance needed in daily living and/or totally dependent.<br>Can't hold objects.
	2	Inability to button shirt but able to eat with a spoon	Power 3/5<br>Marked weakness/stiffness/clumsiness/impairment to daily living.<br>Moderate assistance needed in daily living.<br>Frequently dropping objects.
	3	Able to button a shirt with great difficulty	Power 3/5<br>Moderate weakness/stiffness/clumsiness/impairment to daily living.<br>No/mild assistance needed in daily living.<br>Occasionally drops objects.
	4	Able to button a shirt with slight difficulty	Power 4/5<br>Mild weakness/stiffness/clumsiness/impairment to daily living
	5	No dysfunction	Power 5/5<br>Good strength<br>No weakness
**Sphincter Dysfunction**	0	Inability to urinate voluntarily	Significant/Frequency urinary incontinence<br>Catheter required
	1	Marked difficulty with micturition	Moderate urinary symptoms<br>Moderate impairment to daily living<br>Any episode of urinary incontinence
	2	Mild to moderate difficulty with micturition	Mild urinary symptoms<br>Minor/no impairment to daily living<br>No episodes of incontinence
	3	Normal micturition	No urinary symptoms
**Lower Limb Motor Dysfunction**	0	Complete loss of motor and sensory function	Power 0/5 or 1/5<br>Significant weakness without movement<br>Sensory loss
	1	Sensory preservation without ability to move legs	Power 0/5 or 1/5<br>Significant weakness without movement<br>Preserved sensation
	2	Able to move legs but unable to walk	Power 2/5<br>Significant weakness with minor movement<br>Difficulty in standing<br>Unable to walk
	3	Able to walk on flat floor with a walking aid (i.e. cane or crutch)	Power 3/5<br>Marked weakness/stiffness/clumsiness/impairment to daily living<br>Walking aid
	4	Able to walk up and/or down stairs with a hand rail	Power 3/5<br>Marked weakness/stiffness/clumsiness/impairment to daily living<br>No walking aid
	5	Moderate to significant lack of stability but able to walk up and/or down stairs without hand rail	Power 4/5<br>Mild weakness/stiffness/clumsiness/impairment to daily living<br>Significant imbalance and/or falls
	6	Mild lack of stability but walk unaided with smooth reciprocation	Power 4/5<br>Mild weakness/stiffness/clumsiness/impairment to daily living<br>Some imbalance without falls
	7	No dysfunction	Power 5/5<br>Good strength<br>No weakness
**Sensory Dysfunction**	0	Complete loss of hand sensation	Total loss of hand sensation
	1	Severe sensory loss or pain	Moderate/significant sensory loss<br>Pain more significant than numbness/paraesthesia
	2	Mild sensory loss	Mild/moderate sensory loss/paraesthesia
	3	No sensory loss	Normal sensation

imJOA Development Methodology

1. A translation of mJOA into i-mJOA was designed based upon commonly reported clinical details in patients' electronic records (e.g. "walking aid" vs. "no walking aid" imbalance, or 0-5/5 power grading). This translation is presented in Table 1.
2. The electronic records of 13 patients assessed by a spinal clinic in our centre were collected. These were patients outside of the study range dates and thus were not included in our study. mJOA assessments were made during their clinical consultations and therefore a formal mJOA score was available.
3. These records were anonymised and the mJOA questionnaire results were removed.
4. The mJOA to i-mJOA translation was applied to these patient records.
5. True mJOA and i-mJOA scores were then statistically analysed using the Kappy statistic. The results are presented below.

The imJOA and mJOA had good agreement (0.73), specifically upper limb (0.66), lower limb (0.75), sensory (0.32), sphincter (0.29), and total mJOA (0.73). Average differences between i-mJOA and mJOA ± standard deviation were: upper limb (0.13±0.92), lower limb (0.13±0.92), sensory (-0.4±0.74), sphincter (0.27±0.70), and total mJOA (0.13±1.51).

This experimental tool was applied to assess the disease severity of our cohort. If no features for a subcategory of i-mJOA were documented, the category was assigned the highest score. i-mJOA subcategory scores were assumed to stay constant over time unless specifically mentioned to have

For peer review only

improved or deteriorated. If the criteria were met for more than one score within a subcategory, the lower score was assigned. i-mJOA scores are reported as mean ± standard deviation.

Analysis

Statistical analysis was carried out using SPSS V22 (Chicago, IL, USA). Bivariate analysis was conducted between variables using Pearson's and Spearman's correlations depending on the variable types involved. Wilcoxon signed-rank test was used to calculate significant changes in i-mJOA from primary care referral to surgical assessment. As cases were selected on the basis of having sufficient documentation, there was not missing data.

For peer review only

<u>Results</u>

Study demographics

1123 total scans were screened, resulting in a final cohort of 43 DCM cases (Figure 1). The vast majority of these scans were of unique patients, although rarely patients were re-imaged. The average age at time of symptom onset was 61.4±13.9 years and the majority of patients (28, 65%) were male. Of the 43 cases, 22 (51%) experienced previous myelopathy and the other 21 (49%) were presenting for the first time.

Heterogeneity in primary referrals of DCM patients points to the lack of a unified pathway

33 primary referrals were accessible to review. The majority of these were made

by a general practitioner (28, 85%) and less frequently by a physiotherapist (3, 9%) or other peripheral hospital (2, 6%). Half of all DCM cases were referred directly to a spine surgeon (22, 51%) whilst the other were seen by other specialties first (21, 49%), including neurology (N=16), orthopaedics (N=2), pain management (N=1), rheumatology (N=1) and geriatric medicine (N=1). Almost all cases (42, 98%) were ultimately reviewed by a spinal surgeon. Referral pathways are displayed separating new myelopathy cases from recurrent cases (Figure 2).

DCM patients encounter significant delays from the onset of symptoms to the time of primary referral

Symptom onset was defined as the point at which the patient first experienced symptoms. For example, if a primary referral stated the patient had been experiencing numbness in his fingers for 4 months. The onset of symptoms was defined as 4 months prior to the date of this referral. The times between symptom onset and primary referral for new and recurrent cases of DCM were 8.3±10.1 and 6.4±4.2 months respectively. 76% (16/21) of new cases and 24% (5/22) of recurrent cases of DCM were referred to an alternative specialist for secondary assessment rather than directly to a spine surgeon. The times between primary referral and secondary assessment for new and recurrent cases of DCM were 1.5±0.8 and 1.9±1.2 months respectively.

Secondary referral to spine surgery is consistently delayed and does not take patient characteristics into account

Following secondary assessment, all patients apart from one were then referred

For peer review only

on to see a spine surgeon. The one non-referred patient had a complex neurological and social background with mild myelopathy and was deemed better managed with regular neurology follow-up than consideration of spinal surgery. The times between secondary assessment and surgical assessment for new and recurrent cases of DCM were 5.1±4.0 and 5.5±2.2 months respectively. No clinical feature, patient demographic factor or specialty of secondary assessment was associated with a significantly different speed of referral to spinal surgeon review.

A minority of new DCM patients are directly referred to spine surgery

24% (5/21) of new cases and 77% (17/22) of recurrent cases were referred from primary assessment directly to a spine surgeon. The times between primary assessment and surgical assessment for these new and recurrent cases of DCM were 3.7±4.1 and 0.9±2.2 months respectively. The only factor associated with direct referral to a spinal surgeon was previous DCM ($p<0.001$). The overall times between symptom onset and surgical assessment for new and recurrent cases of DCM were 17.7±16.0 and 9.5±9.0 months respectively. Of the new cases of DCM, 45% (9/20) received an operation, 35% (7/20) received a follow-up appointment with a spine surgeon, and 20% (4/20) were discharged. Of the recurrent cases of DCM, 50% (11/22) received an operation, 23% (5/22) received a follow-up appointment with a spine surgeon, and 27% (6/22) were discharged.

Significant delays in MRI scans relative to symptom onset may contribute to delayed diagnosis of DCM

For peer review only

The observed cohort was formed on the basis of diagnostic cervical MRI scans, demonstrating spinal cord compression. Relative to symptom onset, the scan was performed 17.7±15.6 and 9.5±8.7 months after symptomatic onset for new and recurrent cases of DCM respectively. This scan was performed at 1.4±4.7 and 3.3±4.2 months after secondary assessment for new and recurrent cases of DCM respectively. Relative to surgical review it was performed at 2.3±0.7 months before and 0.5±1.2 months after surgical assessment for new and recurrent cases of DCM respectively. Thus, in general, imaging of new DCM cases was conducted prior to reaching a spinal surgeon, whereas recurrent cases were imaged after initial surgical consultation.

DCM patients continue to deteriorate along the pathway from referral to treatment

Between the earliest recorded assessment (primary or secondary) and surgical assessment, the following deteriorations in i-mJOA scores were observed (Figure 3). 59% of patients deteriorated, 24% remained stable, and 17% showed improvement, presumably due to symptom fluctuation or resolution of disc herniation. The total i-mJOA change for individual patients ranged from +4 to -6 points. On average, patients that deteriorated displayed a reduction of 2.3±1.8 i-mJOA points.

Discussion

The pathway from symptom onset to diagnosis and surgical assessment is complex and highly heterogeneous for DCM patients. They face long delays

For peer review only

before a diagnosis and further delays before surgical assessment. During this time, most experience a progression in symptoms. However, DCM symptoms are by at large not reversible. The observed deterioration is therefore likely to translate into increased long-term disability and lead to lower quality of life.

A diagnosis of DCM is made before reaching a spinal surgeon.

DCM patients principally enter the healthcare system via primary care. They are then referred via another specialty for assessment and imaging, typically Neurology, before reaching a spinal surgeon. This contrasts the study by Behrbalk et al, the only other study to consider the DCM health care system, which found most patients were initially assessed by orthopaedics.[18] A potential explanation is that patients may self-refer to Orthopedics in Israel but require a formal referral to Neurology or Neurosurgery. Regardless, both studies have demonstrated that the diagnosis of new onset DCM is made outside of spinal surgery.

This finding is significant as DCM treatment and research is largely led by spinal surgeons. In order to accelerate diagnosis and improve outcomes based on existing treatment paradigms, greater clarity of the interaction between other specialties and DCM patients is critical.

DCM patients wait months for a diagnosis and years for treatment

Delays in patient pathways were noted in all components of the system. The greatest delay appears to exist between disease onset and primary referral to

For peer review only - http://bmjopen.bmj.com/site/about/guidelines.xhtml

secondary care. This is consistent with Behbalk et al and patient experience.[18]

The cause for this delay is likely to be multi-factorial. Potential factors include late presentation by patients, subtle or non-specific symptoms and examination findings,[24,25] misdiagnoses and a lack of awareness of DCM. Future studies are required to determine the exact reasons for pre-referral delays in order to develop targeted interventions.

Minimising delays of treatment is especially important in DCM, because length of symptoms and disease severity are the two most important factors that determine response to surgery of DCM patients [13-15]. Moreover, shortening the times until patients are seen by a myelopathy specialist is likely to be the single most effective strategy of improving patient outcomes.

The lack of a unified pathway is likely to contribute to delays in treatment

The majority of new cases (76%) were not directly referred to a spine surgeon but instead were reviewed by neurologists, orthopaedics, pain specialists, rheumatologists, and geriatricians. The specialty performing the secondary assessment did not influence referral speed from secondary care to spinal surgery. These findings corroborate that there is no streamlined pathway for surgical assessment of a patient with DCM.

At the early stages, symptoms are often subtle and often missed by non-specialists. However, even asymptomatic patients with MRI-proven cervical spinal cord compression run a considerable risk of developing myelopathy.[28]

For peer review only

A recent study found that 59% of asymptomatic individuals over 40 had cervical cord compression on MRI.[28] A further study observed that 13% of non-myelopathic individuals with cervical cord compression developed myelopathy within a 2-year period.[29]

Our data indicated that disease severity did not influence speed of referral to a spine surgeon. This may seem counter-intuitive but potentially be explained by the broad range of symptoms associated with myelopathy.

For these reasons, the authors advocate more rapid assessment by a specialist service once a patient has proven cord compression, especially alongside clinically myelopathic features. This is also reflected in the first recent international guidelines.[10]

DCM patients continue to deteriorate whilst they wait for treatment

This study found that 59% of patients deteriorated between their earliest available assessment and the point at which they reached assessment by a spine surgeon. The minimum clinically important difference (MCID) in DCM varies by disease severity:[30,31] 1 point for mild disease (mJOA ≥ 15), 2 points for moderate disease (mJOA score of 12–14), and 3 points for severe disease (mJOA score < 12). In the deteriorating group, all but one patient were found to have mild disease (mJOA ≥ 15) at initial assessment. Within this group, the average deterioration was 2.3 i-mJOA points, demonstrating clinically significant disease progression. More rapid DCM referral services and streamlined pathways to specialist assessment could help to prevent this source of potentially non-

For peer review only

reversible deterioration.

What would the ideal pathway be for assessment and management of DCM?

As it stands, this data shows new diagnosis and management as a two-stage process. Stage one: patients receive a diagnosis of DCM outside of spinal surgery based upon clinical symptomatology and MRI findings. Stage two: onward referral to spinal surgery and surgery is offered if indicated.

Whilst direct referral to spinal surgery upon initial clinical suspicion would significantly shorten this process, it would likely lead to a number of problems. Firstly, given the nature of DCM's non-specific symptoms, especially early on, and the limited access in the UK to MRI imaging within primary care, this would lead to many inappropriate referrals and place a strain on already stressed outpatient spinal services. Secondly, these inappropriate referrals are then being assessed by specialists, who can confidently consider DCM, but will not be best placed or experienced to consider other differentials. Therefore, the exclusion of myelopathy could place delays on other diagnoses.

Similar dilemmas have been addressed for many other conditions using intermediate pathways. For example, in the UK, we have a 'musculoskeletal' pathway triaging complaints such as lower back pain and joint pain. In this pathway, patients are referred to physiotherapy led assessment centers, with access to investigations such as MR imaging and electrophysiology. These centers have access to specialist MDTs, who help to decide whether patients can be managed locally or referred onto specialist services. A recent systematic

For peer review only

review has shown such triage was concordant with specialists 68 – 96% of the time and reduced referrals to specialist clinics by 20 – 60%.[26]

At present, the 'musculoskeletal' service is better geared to classically 'orthopaedic' or 'rheumatological' conditions and a potential weakness for DCM would be neurological mimics. In this study, only 9% of referrals came from this musculoskeletal service, with most patients being referred onto Neurology. A more refined assessment process will require further research. Building on the existing intermediate pathway with closer involvement of neurology might be a sensible approach.

For recurrent disease, this study shows that direct access to spinal surgery is appropriate. Of the patients referred, 50% received operations and 23% were kept under surveillance. We speculate that these patients' previous DCM diagnosis heightened awareness by patient and healthcare practitioner, leading to a greater confidence in direct referral to spinal services.

Limitations

Whilst this study was conducted in a single neurosciences centre, it serves a large regional population, almost a tenth of the UK population as a whole.[27] Additionally, given the UK has a National Health Service that delivers care within common structures, the findings in this study are likely generalizable throughout at least the UK health system.

For peer review only

The epidemiology of DCM is poorly characterized, however it is thought to be a common condition.[32] The identification of only 43 cases of DCM over 1 year was lower than expected. However, the majority of patients receiving treatment at this neurosciences centre present with external imaging obtained in referring secondary centres, which did not facilitate access to full clinical records as described in the methodology. Hence, these patients were not included in the present cohort. The low number therefore is likely to reflect the role of the neuroradiology department acting as a secondary centre serving a more local population.

The retrospective nature of the study lead to the development of i-mJOA. While true mJOA would have been preferable, the strong kappa statistics and low average difference indicate that total i-mJOA does represent a reliable proxy and broad-brush method of demonstrating disease severity and changes in disease severity over time retrospectively.

Conclusions

This work demonstrates that patients experience significant delays in diagnosis and time to surgical review. During this time, disease progression occurred and patients functionally deteriorated. The best post-operative outcomes for DCM patients are attained with rapid surgical review and intervention when appropriate. Our findings call for the formation of dedicated streamlined pathways to surgical review for patients with spinal cord compression on MRI, especially in the context of clinical myelopathy. Further prospective system

For peer review only

characterization of factors associated with patient flow is needed to introduce specific targeted interventions to improve the management of DCM in the UK.

Table 1. i-mJOA methodology.

Figure 1. PRISMA flow diagram depicting cohort formation methodology.

Figure 2. Patient referral pathways from symptom onset for recurrent cases of DCM (left) and new cases of DCM (right). Times are given in the format: average months ± standard deviation (number of cases). Dashed lines represent total time durations, not a specific patient pathway.

Figure 3. i-mJOA scores at each clinical phase of assessment. Mean ± standard deviation.

References

1. Nouri A, Tetreault L, Singh A, Karadimas SK, Fehlings MG. Degenerative Cervical Myelopathy: Epidemiology, Genetics, and Pathogenesis. Spine (Phila Pa 1976). 2015 Jun 15;40(12):E675-93

2. Bednarik J, Kadanka Z, Dusek L., et al. Presymptomatic spondylotic cervical myelopathy: an updated predictive model. Eur Spine J. 2008 Mar; 17(3): 421–431.

3. King JTJ, McGinnis KA, Roberts MS. Quality of Life Assessment with the Medical Outcomes Study Short Form-36 among Patients with Cervical Spondylotic Myelopathy. Neurosurgery. 2003 Jan 1;52(1):113.

4. de Oliveira Vilaça C, Orsini M, Leite MA, de Freitas MR, Davidovich E, Fiorelli R, Fiorelli S, Fiorelli C, Oliveira AB, Pessoa BL. Cervical Spondylotic Myelopathy: What the Neurologist Should Know. Neurol Int. 2016 Nov 23;8(4):6330

5. Matz PG, Anderson PA, Holly LT, Groff MW, Heary RF, Kaiser MG, et al.: The natural history of cervical spondylotic myelopathy. J Neurosurg Spine. 2009 Aug;11(2):104-11.

For peer review only

6. Davies, B. M., McHugh, M., Elgheriani, A., Kolias, A. G., Tetreault, L., Hutchinson, P. J. A., et al. The reporting of study and population characteristics in degenerative cervical myelopathy: A systematic review. PloS One, 12(3), e0172564.

7. Fehlings MG, Wilson JR, Kopjar B, et al. Efficacy and safety of surgical decompression in patients with cervical spondylotic myelopathy: results of the AOSpine North America prospective multi-center study. J Bone Joint Surg Am 2013;95:1651–8.

8. Fehlings MG, Ibrahim A, Tetreault L, et al. A global perspective on the outcomes of surgical decompression in patients with cervical spondylotic myelopathy: results from the prospective multicenter AOSpine international study on 479 patients. Spine (Phila Pa 1976). 2015 Sep 1;40(17):1322-8.

9. Oh, T., Lafage, R., Lafage, V., et al. Comparing Quality of Life in Cervical Spondylotic Myelopathy with Other Chronic Debilitating Diseases Using the SF-36 Survey. World Neurosurg. 2017 Oct;106:699-706.

10. Fehlings MG, Tetreault L, Riew K, et al. A Clinical Practice Guideline for the Management of Patients With Degenerative Cervical Myelopathy: Recommendations for Patients With Mild, Moderate, and Severe Disease and Nonmyelopathic Patients With Evidence of Cord Compression. Global Spine Journal. 2017 Sept. 7(3_suppl), 70S–83S.

11. Matz PG, Anderson PA, Groff MW, et al. Cervical laminoplasty for the treatment of cervical degenerative myelopathy. J Neurosurg Spine 11:157–169, 2009

12. Ebersold MJ, Pare MC, Quast LM. Surgical treatment for cervical spondylitic myelopathy. J Neurosurg. 1995 May;82(5):745-51.

13. Holly LT, Matz PG, Anderson PA, et al. Clinical prognostic indicators of surgical outcome in cervical spondylotic myelopathy. J Neurosurg Spine 11:112–118, 2009

14. Zhang JT, Wang LF, Wang S, Li J, Shen Y. Risk factors for poor outcome of surgery for cervical spondylotic myelopathy. Spinal Cord. 2016 Dec;54(12):1127-1131

15. Tetreault L, Kopjar B, Côté P, Arnold P, Fehlings MG. A Clinical Prediction Rule for Functional Outcomes in Patients Undergoing Surgery for Degenerative Cervical Myelopathy: Analysis of an International Prospective Multicenter Data Set of 757 Subjects. J Bone Joint Surg Am. 2015 Dec 16;97(24):2038-46

16. Nouri A, Tetreault L, Côté P, Zamorano JJ, Dalzell K, Fehlings MG. Does Magnetic Resonance Imaging Improve the Predictive Performance of a Validated Clinical Prediction Rule Developed to Evaluate Surgical Outcome in Patients With Degenerative Cervical Myelopathy? Spine (Phila Pa 1976). 2015 Jul

For peer review only

15;40(14):1092-100.

17. “Elemental Ideas - Cervical Spondylotic Myelopathy.” Cambridge TV. www.myelopathy.org, www.cambridge-tv.co.uk/tag/myelopathy/.

18. Behrbalk E, Salame K, Regev GJ, Keynan O, Boszczyk B, Lidar Z. Delayed diagnosis of cervical spondylotic myelopathy by primary care physicians. Neurosurg Focus. 2013 Jul;35(1):E1

19. “Routes to Diagnosis.” Routes to Diagnosis. Macmillan Cancer Support, n.d. eb. 21 May 2017.

20. Buvarp Dyrop H, Vedsted P, Rædkjær M, Safwat A, Keller J. Routes to Diagnosis for Suspected Sarcoma: The Impact of Symptoms and Clinical Findings on the Diagnostic Process. Sarcoma. 2016;2016:8639272. doi: 10.1155/2016/8639272.

21. Walter FM et al. Symptoms and patient factors associated with diagnostic intervals for pancreatic cancer (SYMPTOM pancreatic study): a prospective cohort study. Lancet Gastroenterol Hepatol. 2016 Dec;1(4):298-306.

22. Walter FM et al. Symptoms and patient factors associated with longer time to diagnosis for colorectal cancer: results from a prospective cohort study. Br J Cancer. 2016 Aug 23;115(5):533-41.

23. Davies BM, McHugh M, Elgheriani A, et al. Reported Outcome Measures in Degenerative Cervical Myelopathy: A Systematic Review. PLoS One. 2016 Aug 2;11(8)

24. Rhee JM, Heflin JA, Hamasaki T, Freedman B. Prevalence of physical signs in cervical myelopathy: a prospective, controlled study. Spine (Phila Pa 1976). 2009 Apr 20;34(9):890-5

25. Cook CE, Wilhelm M, Cook AE, Petrosino C, Isaacs R. Clinical tests for screening and diagnosis of cervical spine myelopathy: a systematic review. J Manipulative Physiol Ther. 2011 Oct;34(8):539-46.

26. Hussenbux, A., Morrissey, D., Joseph, C., & McClellan, C. M. Intermediate care pathways for musculoskeletal conditions--are they working? A systematic review. Physiotherapy. 2015 Mar;101(1):13-24.

27. East of England Trauma Network, NHS, www.eoetraumanetwork.nhs.uk/. October 2016.

28. Kovalova I, Kerkovsky M, Kadanka Z, et al. Prevalence and Imaging Characteristics of Nonmyelopathic and Myelopathic Spondylotic Cervical Cord Compression. Spine (Phila Pa 1976). 2016 Dec 15;41(24):1908-1916.

29. Zdenek Kadanka, Jr, Blanka Adamova, Milos Kerkovsky, et al. Predictors of

For peer review only

symptomatic myelopathy in degenerative cervical spinal cord compression. Brain Behav. 2017 Sep; 7(9): e00797.

30. Tetreault L, Nouri A, Kopjar B, Côté P, Fehlings MG: The minimum clinically important difference of the Modified Japanese Orthopaedic Association Scale in patients with degenerative cervical myelopathy. Spine (Phila Pa 1976) 40:1653–1659, 2015

31. Tetreault L, Wilson JR, Kotter MR, et al. Predicting the minimum clinically important difference in patients undergoing surgery for the treatment of degenerative cervical myelopathy. Neurosurg Focus. 2016 Jun;40(6):E14

32. Davies BM, Mowforth OD, Smith EK, Kotter MR. Degenerative cervical myelopathy. BMJ. 2018 Feb 22;360:k186

For peer review only

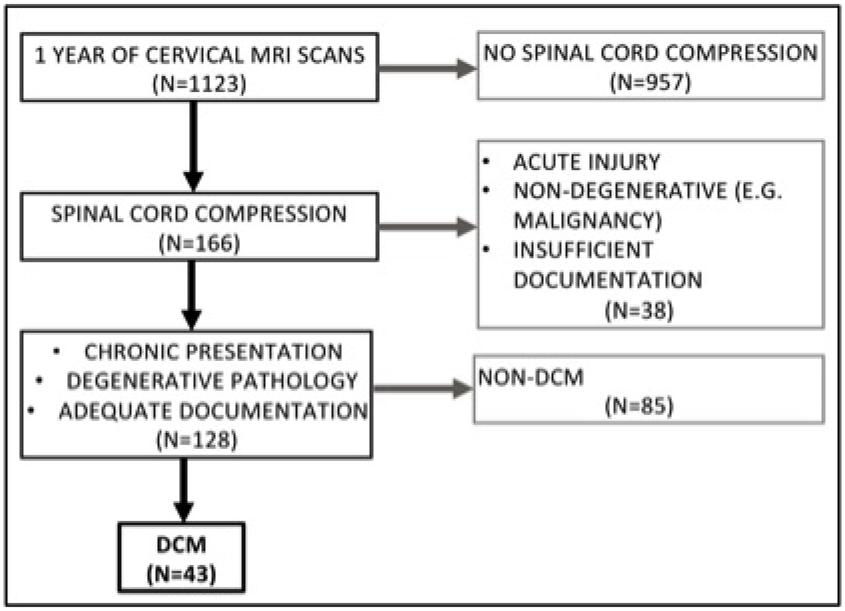

Figure 1. PRISMA flow diagram depicting cohort formation methodology.

35x25mm (300 x 300 DPI)

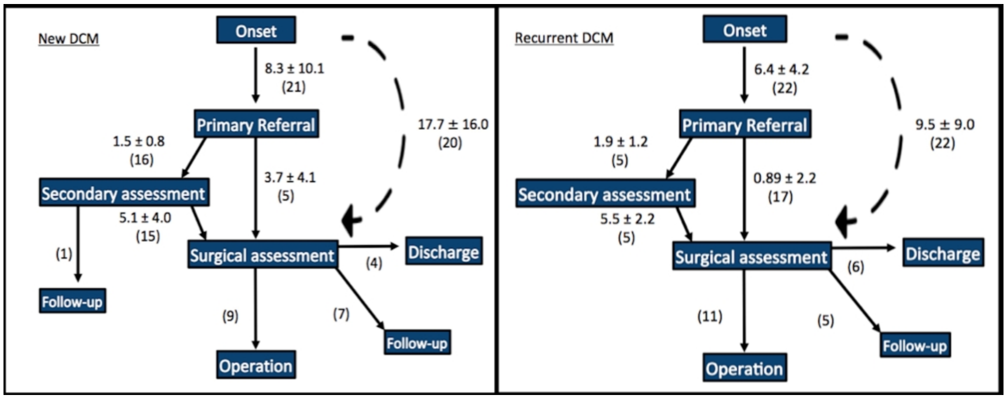

Figure 2. Patient referral pathways from symptom onset for recurrent cases of DCM (left) and new cases of DCM (right). Times are given in the format: average months ± standard deviation (number of cases). Dashed lines represent total time durations, not a specific patient pathway.

426x166mm (300 x 300 DPI)

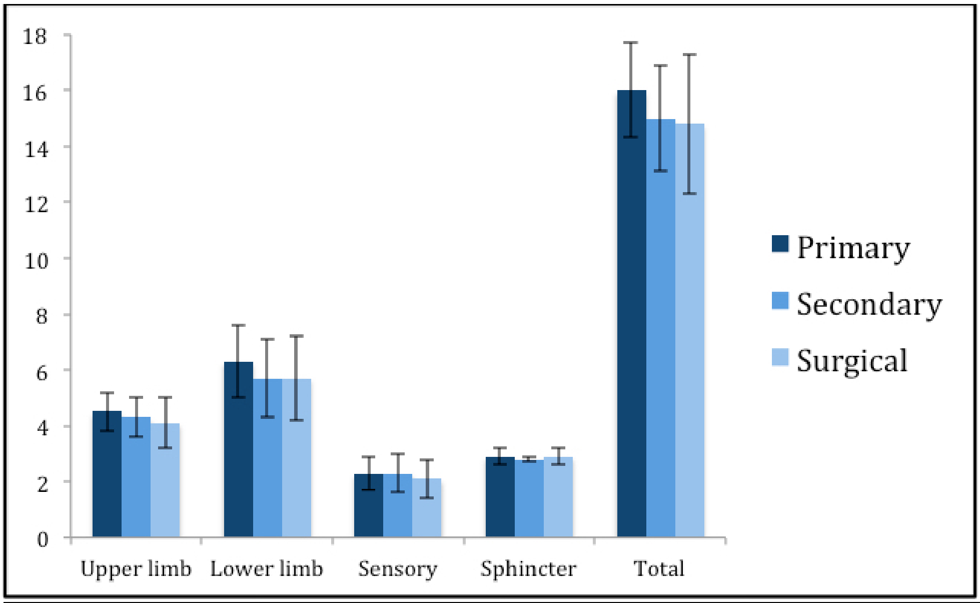

	Primary	Secondary	Surgical
**Upper limb**	4.5±0.7	4.3±0.7	4.1±0.90
**Lower limb**	6.3±1.3	5.7±1.4	5.7±1.5
**Sensory**	2.3±0.6	2.3±0.7	2.1±0.7
**Sphincter**	2.9±0.3	2.8±0.1	2.9±0.3
**Total**	16.0±1.7	15.0±1.9	14.8±2.5

Figure 3. i-mJOA scores at each clinical phase of assessment. Mean ± standard deviation.

55x45mm (300 x 300 DPI)

STROBE Statement

Checklist of items that should be included in reports of observational studies

Section/Topic	Item No	Recommendation	Reported on Page No
**Title and abstract**	1	(*a*) Indicate the study's design with a commonly used term in the title or the abstract	1
		(*b*) Provide in the abstract an informative and balanced summary of what was done and what was found	3
**Introduction**			
Background/rationale	2	Explain the scientific background and rationale for the investigation being reported	5
Objectives	3	State specific objectives, including any prespecified hypotheses	7
**Methods**			
Study design	4	Present key elements of study design early in the paper	7
Setting	5	Describe the setting, locations, and relevant dates, including periods of recruitment, exposure, follow-up, and data collection	7
Participants	6	(*a*) *Cohort study*—Give the eligibility criteria, and the sources and methods of selection of participants. Describe methods of follow-up<br>*Case-control study*—Give the eligibility criteria, and the sources and methods of case ascertainment and control selection. Give the rationale for the choice of cases and controls<br>*Cross-sectional study*—Give the eligibility criteria, and the sources and methods of selection of participants	7
		(*b*) *Cohort study*—For matched studies, give matching criteria and number of exposed and unexposed<br>*Case-control study*—For matched studies, give matching criteria and the number of controls per case	
Variables	7	Clearly define all outcomes, exposures, predictors, potential confounders, and effect modifiers. Give diagnostic criteria, if applicable	8,9
Data sources/measurement	8*	For each variable of interest, give sources of data and details of methods of assessment (measurement). Describe comparability of assessment methods if there is more than one group	8,9
Bias	9	Describe any efforts to address potential sources of bias	10
Study size	10	Explain how the study size was arrived at	11
Quantitative variables	11	Explain how quantitative variables were handled in the analyses. If applicable, describe which groupings were chosen and why	10
Statistical methods	12	(*a*) Describe all statistical methods, including those used to control for confounding	10
		(*b*) Describe any methods used to examine subgroups and interactions	10
		(*c*) Explain how missing data were addressed	10
		(*d*) *Cohort study*—If applicable, explain how loss to follow-up was addressed<br>*Case-control study*—If applicable, explain how matching of cases and controls was addressed<br>*Cross-sectional study*—If applicable, describe analytical methods taking account of sampling strategy	10
		(*e*) Describe any sensitivity analyses	10

For peer review only - http://bmjopen.bmj.com/site/about/guidelines.xhtml

Section/Topic	Item No	Recommendation	Reported on Page No
**Results**			
Participants	13*	(a) Report numbers of individuals at each stage of study—eg numbers potentially eligible, examined for eligibility, confirmed eligible, included in the study, completing follow-up, and analysed	7
		(b) Give reasons for non-participation at each stage	
		(c) Consider use of a flow diagram	
Descriptive data	14*	(a) Give characteristics of study participants (eg demographic, clinical, social) and information on exposures and potential confounders	11
		(b) Indicate number of participants with missing data for each variable of interest	
		(c) *Cohort study*—Summarise follow-up time (eg, average and total amount)	
Outcome data	15*	*Cohort study*—Report numbers of outcome events or summary measures over time	
		Case-control study—Report numbers in each exposure category, or summary measures of exposure	
		Cross-sectional study—Report numbers of outcome events or summary measures	11
Main results	16	(*a*) Give unadjusted estimates and, if applicable, confounder-adjusted estimates and their precision (eg, 95% confidence interval). Make clear which confounders were adjusted for and why they were included	11-18
		(*b*) Report category boundaries when continuous variables were categorized	
		(*c*) If relevant, consider translating estimates of relative risk into absolute risk for a meaningful time period	
Other analyses	17	Report other analyses done—eg analyses of subgroups and interactions, and sensitivity analyses	9
**Discussion**			
Key results	18	Summarise key results with reference to study objectives	20
Limitations	19	Discuss limitations of the study, taking into account sources of potential bias or imprecision. Discuss both direction and magnitude of any potential bias	19
Interpretation	20	Give a cautious overall interpretation of results considering objectives, limitations, multiplicity of analyses, results from similar studies, and other relevant evidence	20
Generalisability	21	Discuss the generalisability (external validity) of the study results	19
**Other Information**			
Funding	22	Give the source of funding and the role of the funders for the present study and, if applicable, for the original study on which the present article is based	1

**Give information separately for cases and controls in case-control studies and, if applicable, for exposed and unexposed groups in cohort and cross-sectional studies.*

Note: An Explanation and Elaboration article discusses each checklist item and gives methodological background and published examples of transparent reporting. The STROBE checklist is best used in conjunction with this article (freely available on the Web sites of PLoS Medicine at http://www.plosmedicine.org/, Annals of Internal Medicine at http://www.annals.org/, and Epidemiology at http://www.epidem.com/). Information on the STROBE Initiative is available at www.strobe-statement.org.

For peer review only - http://bmjopen.bmj.com/site/about/guidelines.xhtml

University of Cambridge
Anne McLaren Laboratory for Regenerative Medicine
Department of Clinical Neurosciences
WT MRC Cambridge Stem Cell Institute
West Forvie Building, Forvie Site
CB2 0SZ, Cambridge

Telephone: +44 1223 763366
Fax: +44 1223 763350

Dr Mark R Kotter MD MPhil PhD (cantab)
NIHR Clinician Scientist

Direct line: +44 1223 747476
E-mail: mrk25@cam.ac.uk

01/10/2018

Dr Anna Clark
Assistant Editor
BMJ Open

Re: Manuscript – "Delayed diagnosis in Degenerative Cervical Myelopathy: an overview of patient flow in a UK healthcare system"

Thank you for re-considering your decision on the aforementioned manuscript.

We found the reviewer's comments to be very constructive and helpful. Overall, we were pleased to see that the reviewers found the work to be **"interesting"**, **"well-designed"** and **"well written"**. The panel agree that the research demonstrates diagnostic delays in DCM that may be amenable to targeted interventions in order to improve patient outcomes.

It is worth noting that whilst anecdotally diagnostic delay is commonly reported in DCM, there has only previously been one study, which looked at 42 patients retrospectively (Behrbalk et al, 2013). Our study extends this previous work by constructing patient flow pathways and incorporating time intervals between points of healthcare contact. Our study is also set in the context of the UK heathcare system, in contrast to Behrbalk et al. who carried out their work in Israel, where patients can self-refer to orthopaedics, thus drastically altering specialist access by patients. Our study also uniquely offers insight into disease progression during lag time in diagnosis, a previously unreported process. Given the recognised impact of diagnostic delay on patient outcomes (Tetreault et al, 2015), this is an important area of management to further investigate in order to design improvement strategies.

We feel that by addressing the points raised by the reviewers our manuscript has gained gain further strength and clarity and is an important contribution for the field.

For peer review only

For peer review only - http://bmjopen.bmj.com/site/about/guidelines.xhtml

Please find our responses collated below regarding amendments that have been. The comments of each reviewer are addressed in turn. Every comment is addressed and major changes to the text of the article related to any given point raised by a reviewer are also present in this document in red text for ease of reference. A highlighted version of the revised manuscript will also be uploaded for additional reference.

We hope you are willing to consider our revised manuscript for publication.

Yours faithfully,

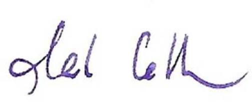

Behrbalk E, Salame K, Regev GJ, Keynan O, Boszczyk B, Lidar Z. Delayed diagnosis of cervical spondylotic myelopathy by primary care physicians. Neurosurg Focus. 2013 Jul;35(1):E1

Tetreault L, Kopjar B, Côté P, Arnold P, Fehlings MG. A Clinical Prediction Rule for Functional Outcomes in Patients Undergoing Surgery for Degenerative Cervical Myelopathy: Analysis of an International Prospective Multicenter Data Set of 757 Subjects. J Bone Joint Surg Am. 2015 Dec 16;97(24):2038-46

For peer review only

Reviewer 1

Dear Andrei F Joaquim,

Thank you kindly for your review of our article. We very much appreciate your feedback.

1.1 You "recommend to evaluate and present more data about delay in CDD already reported in literature".

To the authors' knowledge, we have presented the extent of literature surrounding diagnostic delays in DCM. The single paper that has been previously published focusing on this issue was Behrbalk et al 2013, which we cover in our Discussion section. It is worth noting that these authors cited *"they have found no published data on the process and time frame involved in attaining a diagnosis of CSM".* There has certainly been nothing published since.

We suspect that your comment likely reflects that delays in diagnosis are well recognised anecdotally amongst spinal surgeons and patients. We have found through feedback and discussion within two conferences in the UK and one in Europe at which this research was presented that diagnostic delays are a well-recognised but almost entirely unreported phenomenon. Understanding and improving diagnostic delays are also a significant priority for patients; this was noted by a forum of patients from Myelopathy.org ("Elemental Ideas").

Behrbalk E, Salame K, Regev GJ, Keynan O, Boszczyk B, Lidar Z. Delayed diagnosis of cervical spondylotic myelopathy by primary care physicians. Neurosurg Focus. 2013 Jul;35(1):E1

"Elemental Ideas - Cervical Spondylotic Myelopathy." Cambridge TV. www.myelopathy.org, www.cambridge-tv.co.uk/tag/myelopathy/.

Conferences:

Joint meeting between the German Society of Neurosurgery and the Society of British Neurological Surgeons 2017 - Madgeburg, Germany.

Society of British Neurological Surgeons Autumn Conference 2017 – Liverpool, UK.

Society of British Neurological Surgeons Spring Conference 2016 – Oxford, UK.

Reviewer 2

Dear Paul C. Willems, MD, PhD,

Thank you kindly for your review of our article. We appreciate your feedback and hope you find each of your issues raised adequately addressed below.

2.1 Issue 1:
You highlight a "selection bias" introduced by the fact that the authors chose to exclude patients who had their MRI scan conducted in peripheral hospitals and were subsequently referred on to our tertiary neurosciences centre for further specialist management. You did not feel that this limitation was sufficiently addressed in our manuscript. This is a helpful observation and we appreciate the chance to clarify our methodology.

The goal of this research was to track patients from onset of symptomatic myelopathy to the point at which they were assessed by a spinal surgeon for consideration of surgery. Peripheral hospitals in our region use different electronic and paper record keeping systems from one another and from our centre. Therefore, inclusion of peripherally referred patients would leave us only with their MRI scan imaging and report and the clinical record of their assessment by one of the spine surgeons working in our centre. This would not provide sufficient data to track their disease course and referral pathway.

As well as acting as a tertiary referral centre, our hospital also acts as a large regional hospital serving a diverse and sizeable local population. Therefore, inclusion of only patients imaged at our centre facilitated access to all records of these patients and proper documentation of their disease journeys.

We have clarified this point in our cohort description in our Methods section.

"Peripheral hospitals in our region use different electronic and paper record keeping systems from one another and from this tertiary centre. Therefore, this study only included patients imaged in our centre, which acts as a large regional hospital serving a sizeable and diverse population, as this permitted full access to clinical documentation required to accurately document patient pathways. "

2.2 Issue 2:

For peer review only - http://bmjopen.bmj.com/site/about/guidelines.xhtml

You raised concern over the use of our inferred mJOA (i-mJOA) scoring system: i-mJOA is “retrospectively based on reports from physicians/family doctors which makes it highly susceptible for bias”, “I think this validation should be reported much more in detail, preferably performed by others, and it should be acknowledged much more clearly that this is a serious limitation of the study.”

The authors fully acknowledge the limitation of the i-mJOA scoring and will further elaborate on its development and limitations.

Rationale for Development / Intended Use
It was developed as a research tool for this study to allow retrospective assessment of change in DCM disability, as grading tools are not routinely employed in clinical consultations. It was not specifically developed for wider usage (which would clearly require external validation). The authors wished to demonstrate that neurological and functional deterioration occurs over the delayed time to diagnosis, not that an absolute value or rate for deterioration could be determined.

Development Methodology
The authors appreciate that our methodology for design of i-mJOA was not fully described in our manuscript. We are of course happy to provide further details:
1. Author BH designed a translation of mJOA into i-mJOA based upon commonly reported clinical details in patients' electronic records (e.g. “walking aid” vs. “no walking aid” imbalance, or 0-5/5 power grading). This translation is presented in Table 1. Other similar translations of the mJOA score have previously been conducted. Most recently the P-mJOA was created for ease of patient use and was validated against true mJOA scores with similar results to our i-mJOA (Rhee et al. 2018).
2. The electronic records of 13 patients assessed by a spinal clinic in our centre were collected by author JTM. These were patients outside of the study range dates and thus were not included in our study. mJOA assessments were made during their consultation and therefore a formal mJOA score was available.
3. These records were then anonymised by JTM of any identifying details and the mJOA questionnaire results were removed.
4. Author BH then applied the mJOA to i-mJOA translation to these patient records.
5. True mJOA and i-mJOA scores were then statistically analysed for validation and these results are presented in the manuscript.
6. Total mJOA and total i-mJOA scores showed very close correlation, 0.13±1.51 (kappa 0.73). Thus, the authors felt that the i-mJOA was a suitable tool to apply for this study in order to capture the neurological deterioration that was evident in patients clinical records.

Once again, i-mJOA was not intended to be a perfectly accurate tool for retrospectively creating mJOA scores, but rather a broad-brush method of demonstrating disease severity and changes in disease severity over time retrospectively. Therefore, whilst acknowledging its clear limitations, the authors do not feel i-mJOA constitutes a methodological problem.

Additions to text of article:

“The mJOA is a common and well-validated assessment of disability in DCM.[10,23]

For peer review only

A retrospective method for assessment of the patient's modified Japanese Orthopaedic Association [mJOA] score was developed (Table 1) and prospectively validated (N=13) using a Kappa statistic. This was termed the inferred mJOA [i-mJOA]. imJOA was developed as a research tool for this study to allow retrospective assessment of change in DCM disability, as grading tools are not routinely employed in clinical consultations. It was not specifically developed for wider usage, which would require thorough external validation prior to application.

imJOA Development Methodology

1. A translation of mJOA into i-mJOA was designed based upon commonly reported clinical details in patients' electronic records (e.g. "walking aid" vs. "no walking aid" imbalance, or 0-5/5 power grading). This translation is presented in Table 1.
2. The electronic records of 13 patients assessed by a spinal clinic in our centre were collected. These were patients outside of the study range dates and thus were not included in our study. mJOA assessments were made during their clinical consultations and therefore a formal mJOA score was available.
3. These records were anonymised and the mJOA questionnaire results were removed.
4. The mJOA to i-mJOA translation was applied to these patient records.
5. True mJOA and i-mJOA scores were then statistically analysed using the Kappa statistic. The results are presented below. "

We hope that our clarifications above as well as the corresponding additions to the text of our article (Methods and Limitations sections) fully address your concerns.

Many thanks again for your input. We feel that the article has gained strength and clarity through these amendments.

Rhee JM, Shi WJ, Cyriac M et al. The P-mJOA: A Patient-derived, Self-reported Outcome Instrument for Evaluating Cervical Myelopathy: Comparison with the mJOA. Clin Spine Surg. 2018 Mar;31(2):E115-E120.

Reviewer 3

Dear Catherine Jutzeler,

Thank you for your thorough review of our article. Please find each point individually addressed below.

3.1 Text:

1) The introduction is very lengthy and could be shorten (e.g., part about epidemiology and symptoms)

The authors agree that the introduction is detailed. The purpose of our lengthy introduction to DCM was that we hope this paper will reach an audience extending beyond the spinal community (e.g. primary care) who may need further background on the topic of DCM especially in terms of its epidemiology.

2) The authors should state the design of their epidemiological study (retrospective and observational) at the beginning of the method section.

The authors have made this addition.

“ ***Study description***

Restrospective, observational cohort study”

3) If the study was registered at clinicaltrial.gov, please provide the NTC identifier.

For peer review only

The study was not registered at clinicaltrial.gov. The data was collected as part of a broad service evaluation project.

4) Please specify who screened the radiological reports.

The radiological reports were all read and screened by author BH.

5) How was DCM defined? Was the mJOA a part of the assessment? If not, please explain.

DCM was defined as spinal cord compression on MRI and the presence of a clinical diagnosis of myelopathy in the patient's clinical records. The authors, however, were not defining DCM per se. The final stage of our cohort formation was to examine clinical records for previous clinicians having made a diagnosis of DCM, which would have been based upon the above definition.
mJOA was unfortunately not part of most assessments as it is not routinely calculated during consultations, especially across specialities. Thus, we designed the i-mJOA to serve by proxy in its place as a gross marker for neurological disability and disease severity.

6) As the onset of DCM occurs often before the patient presents to a physician, I am wondering how the authors define the age at time of onset? Or do the authors refer to the age the patient was diagnosed? In that case, it might be better to talk about age of diagnosis?

The time of onset was calculated by examining the referral letter from primary care to secondary care. Each letter stated a time frame for symptoms. E.g. Mr X has been suffering from imbalance and difficulties with his dexterity for 3 months". 3 months prior to the date of this referral was taken to be the onset of symptoms. The authors have clarified this point in the manuscript.

"Symptom onset was defined as the point at which the patient first experienced symptoms. For example, if a primary referral stated the patient had been

For peer review only

experiencing numbness in his fingers for 4 months. The onset of symptoms was defined as 4 months prior to the date of this referral."

7) In the results section and figure 1, the authors state: '1123 scans were screened, resulting in a final cohort of 43 DCM cases'. Were these scans of 1123 patients? Or 1123 scans of which some are from the same patient? Please clarify this as this might be not clear to readers who are not experts in the field.

1123 was the number of scan reports reviewed. Rarely patients were re-imaged within the study time frame. The authors have amended this for clarification.

"1123 total scans were screened, resulting in a final cohort of 43 DCM cases (Figure 1). The vast majority of these scans were of unique patients, although rarely patients were re-imaged. "

8) In the chart flow (figure 1) it is indicated that 85 patients with cervical spinal cord compression were excluded due to no DCM. Did the authors follow up on these patients to assess how many of these patients have developed a DCM later?

No, the authors did not follow these patients up. Only a small number of these would be expected to become myelopathic within the time duration of this study. Furthermore, given that the goal of the study was to examine patients from symptom onset to spinal surgeon review, asymptomatic patients with spinal cord compression would most likely experience different diagnostic pathways and it would be a distinct but important project in its own right to follow these patients up over time.

9) The authors should also fill in the STROBE checklist and submit with the manuscript

The authors will gladly provide a STROBE checklist as part of the submission of a revised manuscript.

3.2 Table:

The authors are encouraged to provide a table with individual data: demographics, i-mJOA scores at each clinical phase assessment (i.e., Figure 3 should then be

removed), radiological findings, initials symptoms, clinical and radiological evidence of the deterioration whilst patients wait for treatment. This information would be very helpful for the reader to interpret the data and results.

The authors did originally consider producing such a table but given the sheer volume of information such a table would need to include to completely depict the cases of 43 patients, we felt this would require a relatively large and inaccessible table.

3.3 Figure 2:

1) It is not clear what the difference between ‘regular follow-up’ and ‘follow-up’ is. Please clarify.

Thank you for pointing this out. It was written in error. ‘regular follow-up’ simply referred to follow-up outside of spinal surgical services (e.g. by a neurologist in this case). The authors will amend the figure to clarify.

See Figure 2 for amendment

2) Please explain in the figure legend what the dashed lines mean.

The dashed lines depict total time durations for ease of visualisation, not a specific patient pathway. The authors will clarify this in the figure legend.

See Figure 2 legend for amendment

3) Is there a reason why ‘regular follow-up’ is not in a blue box?

No, the authors will correct this and clarify as described above.

See Figure 2 for amendment

For peer review only

4) Please define onset. From this figure it is not clear if the onset of DCM is patient reported (i.e., when the patient first noticed symptoms) or refers to the first time the patient was seen by a physician or physical therapist?

Onset refers to the patient-experienced symptom onset. The authors have clarified this in the legend of the figure and the text of the article.

See Figure 2 legend for ammendment

5) Who performed the secondary assessment? Spine surgeons? Or other specialists?

Secondary assessment was performed by a range of non-spine surgeon specialists as described in the main body of the manuscript: neurology (N=16), orthopaedics (N=2; non-spinal orthopaedics), pain management (N=1), rheumatology (N=1) and geriatric medicine (N=1). This is described in the Results section "*Heterogeneity in primary referrals of DCM patients points to the lack of a unified pathway.*" *(page 11)*

6) The figure quality (it is blurry and difficult to interpret) appears to be low. This could be due to the PDF version this reviewer has access to. But it might be worth it to double check.

We apologise for this issue. The image met the specifications set by the journal. Perhaps an issue has arisen with the PDF version. We have re-formatted the image with the above corrections. It should now be clearer to view. Thank you for pointing this out.

Many thanks for your detailed feedback. We feel that the article has gained both clarity and strength for making the subsequent amendments.

For peer review only

BMJ Open

Route to diagnosis of degenerative cervical myelopathy in a UK healthcare system: a retrospective cohort study

Journal:	*BMJ Open*
Manuscript ID	bmjopen-2018-027000.R1
Article Type:	Research
Date Submitted by the Author:	11-Nov-2018
Complete List of Authors:	Hilton, Bryn; University of Cambridge<br>Tempest-Mitchell, Jennifer; University of Cambridge<br>Davies, Benjamin; University of Cambridge, Department of Clinical Neurosurgery<br>Kotter, Mark; University of Cambridge, Department of Clinical Neurosurgery; Ann McLaren Laboratory of Regenerative Medicine, Department of Clinical Neurosciences
**Primary Subject Heading**:	Neurology
Secondary Subject Heading:	Diagnostics, Surgery
Keywords:	Spine < ORTHOPAEDIC & TRAUMA SURGERY, Neurological injury < NEUROLOGY, NEUROLOGY

SCHOLARONE™ Manuscripts

For peer review only - http://bmjopen.bmj.com/site/about/guidelines.xhtml

Route to Diagnosis of Degenerative Cervical Myelopathy in a UK healthcare system: a retrospective cohort study

Bryn Hilton[1], Jennifer Tempest-Mitchell[1], Benjamin M. Davies M.R.C.S B.Sc[2], Mark R.N. Kotter M.D. M.Phil. Ph.D. [2-3]

1) School of Clinical Medicine, University of Cambridge, Cambridge, UK
2) Academic Neurosurgery Unit, Department of Clinical Neurosurgery, University of Cambridge, Cambridge, UK
3) WT MRC Cambridge Stem Cell Institute, Anne McLaren Laboratory, University of Cambridge, UK

For peer review only

Key words:
Cervical, Myelopathy, Spondylosis, Degeneration

Word Count: *3008*

Reference Count: *32*

Acknowledgements:

Research in the senior author's laboratory is supported by a core support grant from the Wellcome Trust and MRC to the Wellcome Trust-Medical Research Council Cambridge Stem Cell Institute. MRNK is supported by a NIHR Clinician Scientist Award.

Disclaimer: This report is independent research arising from a Clinician Scientist Award, CS-2015-15-023, supported by the National Institute for Health Research. The views expressed in this publication are those of the authors and not necessarily those of the NHS, the National Institute for Health Research or the Department of Health.

Contributorship Statement

BH – study design, data collection, data analysis, manuscript writing.

JTM – data collection, manuscript editing.

BD – study design, manuscript editing.

MK – study design, manuscript editing.

Ethics Declarations
All data was collected as part of a service evaluation and appropriate prior permission was sought and granted.

Data Sharing Statement
No additional data available

Competing Interests Statement
There are no competing interests for any authors involved in this publication.

Correspondence:
Dr. Mark R.N. Kotter

Department of Clinical Neurosciences
Ann McLaren Laboratory of Regenerative Medicine
University of Cambridge
West Forvie Building
Forvie Site Box 213
Hills Road
Cambridge
CB2 0SZ
UK

Email: mrk25@cam.ac.uk
Work telephone: 01223 747476
Work fax: 01223 763350

For peer review only

Objectives
Degenerative Cervical Myelopathy (DCM) presents insidiously, making initial diagnosis challenging. Surgery has been shown to prevent further disability but existing spinal cord damage may be permanent. Delays in surgery lead to increased disability and reduced post-operative improvements. Therefore, rapid surgical assessment is key to improving patient outcomes. Unfortunately, diagnosis of DCM in primary care is often delayed. This study aimed to characterise DCM patients' route to diagnosis and surgical assessment as well as to plot disease progression over time.

Design
Restrospective, observational cohort study

Setting
Single, tertiary centre using additional clinical records from primary and secondary care centres.

Participants
One year of cervical MRI scans conducted at a tertiary neurosciences centre (N=1123) were screened for cervical cord compression, a corresponding clinical diagnosis of myelopathy, and sufficient clinical documentation to plot a route to diagnosis (N=43).

Primary outcome measures
Time to diagnosis from symptom onset, route to diagnosis, and disease progression were the primary outcome measures in this study. Disease severity was approximated using a prospectively validated method for inferring modified Japanese Orthopaedic Association (i-mJOA) functional scoring from clinical documentation.

Results
Patients received a referral to secondary care 6.4±7.7 months after symptom onset. Cervical MRI scanning and neurosurgical review occurred 12.5±13.0 and 15.8±13.5 months after symptom onset respectively. i-mJOA was 16.0±1.7 at primary care assessment and 14.8±2.5 at surgical assessment. 61.0% of patients were offered operations. For those who received surgery, time between onset and surgery was 22.1±13.2 months.

Conclusions
Route to surgical assessment was heterogeneous and lengthy. Some patients deteriorated during this period. This study highlights the need for a streamlined pathway by which patients with cervical cord compression can receive timely assessment and treatment by a specialist. This would improve outcomes for

For peer review only

patients using existing treatments.

Strengths and Limitations of this Study

·This is the largest study to characterise DCM patient flow and the first to do so within the UK healthcare system.
·Centralized, electronic case notes permitted accurate construction of patient flow pathways throughout the healthcare system.
·Sparse clinical records lead to the exclusion of some cases that would otherwise have been suitable and thus decreased sample size.
·This study was conducted in a single-centre and thus further work is required to generalise conclusions.
·True m-JOA scores were not available and thus i-mJOA was developed to retrospectively assess disease severity from case notes.

For peer review only

Introduction

Degenerative Cervical Myelopathy (DCM) is an umbrella term for compression of the cervical spinal cord due to degenerative pathology, such as osteophyte formation, intervertebral disc protrusion, and ligament hypertrophy or ossification[1]. The estimated minimum incidence and prevalence of DCM's are 41 and 605 per 1,000,000 respectively in North America[1]. However, this is likely to be an underestimate as DCM often goes undiagnosed and epidemiology frequently relies on operative incidence.[1] In a recent study of 183 randomly selected adults aged 40-80, 59% had radiological evidence of cervical cord compression and 1% as yet undiagnosed DCM. The evolution of incidental cord compression is unclear, but in the only study of its kind Bednarik et al demonstrated 23% went onto develop DCM in a series of 199 patients.[2] These findings would suggest DCM is far more common than currently demonstrated.

DCM causes progressive neurological dysfunction leading to significant disability and reduction in quality of life for patients.[3] Symptoms include limb spasticity, numb and clumsy hands, sphincter dysfunction, neck and limb pain, imbalance, and limb weakness leading to falls.[4] These symptoms often arise and progress insidiously in either a step-wise or continuous manner.[5] The rate of progression varies between individuals and cannot currently be predicted.

A diagnosis of DCM requires congruent symptoms, clinical and MRI findings, typically evidence of spinal cord compression.[6] Surgery is the only evidence based treatment for DCM and has been shown to halt disease progression and

For peer review only

afford some improvement across a number of domains.[5,7,8] However few patients make a complete recovery. This is due to the limited regenerative capacity of the spinal cord; as a result existing damage is often permanent and leads to life-long disabilities. A recent study has demonstrated that DCM patients have amongst the lowest SF-36 scores of chronic diseases.[9] Therefore improving functional recovery in DCM is a major unmet clinical need.

Recent international guidelines advise all DCM patients should be assessed by a specialist service and surgery offered for moderate to severe disease, as well as any progressive disease.[10]

Prognostic factors for post-operative functional improvement have been extensively studied. The main predictor of outcome is pre-surgical morbidity. Detecting progressive disease and early intervention is therefore important. Moreover, longer pre-operative symptom duration is associated with poorer post-operative improvement.[11-16] In the largest cohort study of DCM patients, surgery within 6 months of symptom onset offered the best chance of recovery. In this context, rapid referral to a spinal surgeon followed by prompt surgical intervention when appropriate is key to minimising disability.

Unfortunately, DCM patients report significant delays in diagnosis.[17] This is corroborated in the study of Behrbalk et al (2013) who found that most patients in Israel wait more than two years for a diagnosis.[18] If this could be overcome, outcomes would improve based on existing treatment strategies. Whilst the delay in diagnosis is becoming better recognised, where and why the delays

For peer review only

occur remain unclear.

Healthcare system research aims to characterise patient pathways, to identify the 'where' and 'why' of delays, to allow targeted logistical interventions. This process has been successful in cancer presentation and management,[19-22] informing clinician- and public policy-based initiatives to improve cancer care.

Our objectives, therefore, were to characterize the DCM patient pathways within the UK healthcare system in order to support targeted interventions aimed at accelerating diagnosis and management. This is the first study of its kind in the field of DCM.

Methods

Study description

Restrospective, observational cohort study

Cohort Description

The radiological report of all cervical spine MRI scans conducted over one year in a single, tertiary neurosciences centre were screened for descriptions of cervical spinal cord compression. The centre serves an estimated population of 5.9 million, although a considerable proportion of spinal patients are initially seen and are imaged, and may be managed in secondary care centres.[22] The clinical records corresponding to these scans were examined and cross-matched against inclusion criteria: non-traumatic cord compression due to spinal degeneration, a clinical diagnosis of DCM and adequate clinical documentation to characterise

For peer review only

the referral pathway. Cases that met these criteria formed the cohort for this study (Figure 1).

Peripheral hospitals in our region use different electronic and paper record keeping systems from one another and from this tertiary centre. Therefore, this study only included patients imaged in our centre, as this permitted full access to clinical documentation required to accurately document patient pathways.

Patient and Public Involvement

This study was developed in response to patient reports of frequent and long delays in their diagnosis and management. These reports came from the community of myelopathy.org, an international DCM charity and were reconfirmed as a priority research area during a Patient and Public Involvement day at the University of Cambridge. Some of these interviews were featured in a video documentary for Cambridge TV. While patients drove the theme for the study through their testimonials, they were not involved in the exact design of the study, which was informed by local referral pathways and literature on DCM. Once published, this study will be shared through myelopathy.org to make its findings widely available to DCM patients and carers.

Data Collection

All relevant clinical records (assessments, correspondences, inpatient documentation) were examined for the cohort cases. Demographic data was collected: age at time of onset, sex, relevant commodities, previous episodes of DCM and previous cervical surgery.

For peer review only

Retrospective Assessment of Disease Severity

The mJOA is a common and well-validated assessment of disability in DCM.[10,23] A retrospective method for assessment of the patient's modified Japanese Orthopaedic Association [mJOA] score was developed (Table 1) and prospectively validated (N=13) using a Kappa statistic. This was termed the inferred mJOA [i-mJOA]. imJOA was developed as a research tool for this study to allow retrospective assessment of change in DCM disability, as grading tools are not routinely employed in clinical consultations. It was not specifically developed for wider usage, which would require thorough external validation prior to application.

For peer review only

Table 1.

	Score	mJOA	imJOA
**Upper Limb Motor Dysfunction**	0	Inability to move hands	Power 0/5 or 1/5<br>No movement
	1	Inability to eat with a spoon but able to move hands	Power 2/5<br>Significant weakness/stiffness/clumsiness/impairment to daily living.<br>Significant assistance needed in daily living and/or totally dependent.<br>Can't hold objects.
	2	Inability to button shirt but able to eat with a spoon	Power 3/5<br>Marked weakness/stiffness/clumsiness/impairment to daily living.<br>Moderate assistance needed in daily living.<br>Frequently dropping objects.
	3	Able to button a shirt with great difficulty	Power 3/5<br>Moderate weakness/stiffness/clumsiness/impairment to daily living.<br>No/mild assistance needed in daily living.<br>Occasionally drops objects.
	4	Able to button a shirt with slight difficulty	Power 4/5<br>Mild weakness/stiffness/clumsiness/impairment to daily living
	5	No dysfunction	Power 5/5<br>Good strength<br>No weakness
**Sphincter Dysfunction**	0	Inability to urinate voluntarily	Significant/Frequency urinary incontinence<br>Catheter required
	1	Marked difficulty with micturition	Moderate urinary symptoms<br>Moderate impairment to daily living<br>Any episode of urinary incontinence
	2	Mild to moderate difficulty with micturition	Mild urinary symptoms<br>Minor/no impairment to daily living<br>No episodes of incontinence
	3	Normal micturition	No urinary symptoms
**Lower Limb Motor Dysfunction**	0	Complete loss of motor and sensory function	Power 0/5 or 1/5<br>Significant weakness without movement<br>Sensory loss
	1	Sensory preservation without ability to move legs	Power 0/5 or 1/5<br>Significant weakness without movement<br>Preserved sensation
	2	Able to move legs but unable to walk	Power 2/5<br>Significant weakness with minor movement<br>Difficulty in standing<br>Unable to walk
	3	Able to walk on flat floor with a walking aid (i.e. cane or crutch)	Power 3/5<br>Marked weakness/stiffness/clumsiness/impairment to daily living<br>Walking aid
	4	Able to walk up and/or down stairs with a hand rail	Power 3/5<br>Marked weakness/stiffness/clumsiness/impairment to daily living<br>No walking aid
	5	Moderate to significant lack of stability but able to walk up and/or down stairs without hand rail	Power 4/5<br>Mild weakness/stiffness/clumsiness/impairment to daily living<br>Significant imbalance and/or falls
	6	Mild lack of stability but walk unaided with smooth reciprocation	Power 4/5<br>Mild weakness/stiffness/clumsiness/impairment to daily living<br>Some imbalance without falls
	7	No dysfunction	Power 5/5<br>Good strength<br>No weakness
**Sensory Dysfunction**	0	Complete loss of hand sensation	Total loss of hand sensation
	1	Severe sensory loss or pain	Moderate/significant sensory loss<br>Pain more significant than numbness/paraesthesia
	2	Mild sensory loss	Mild/moderate sensory loss/paraesthesia
	3	No sensory loss	Normal sensation

imJOA Development Methodology

1. A translation of mJOA into i-mJOA was designed based upon commonly reported clinical details in patients' electronic records (e.g. "walking aid" vs. "no walking aid" imbalance, or 0-5/5 power grading). This translation is presented in Table 1.
2. The electronic records of 13 patients assessed by a spinal clinic in our centre were collected. These were patients outside of the study range dates and thus were not included in our study. mJOA assessments were made during their clinical consultations and therefore a formal mJOA score was available.
3. These records were anonymised and the mJOA questionnaire results were removed.
4. The mJOA to i-mJOA translation was applied to these patient records.
5. True mJOA and i-mJOA scores were then statistically analysed using the Kappy statistic. The results are presented below.

The imJOA and mJOA had good agreement (0.73), specifically upper limb (0.66), lower limb (0.75), sensory (0.32), sphincter (0.29), and total mJOA (0.73). Average differences between i-mJOA and mJOA ± standard deviation were: upper limb (0.13±0.92), lower limb (0.13±0.92), sensory (-0.4±0.74), sphincter (0.27±0.70), and total mJOA (0.13±1.51).

This experimental tool was applied to assess the disease severity of our cohort. If no features for a subcategory of i-mJOA were documented, the category was assigned the highest score. i-mJOA subcategory scores were assumed to stay constant over time unless specifically mentioned to have

For peer review only

improved or deteriorated. If the criteria were met for more than one score within a subcategory, the lower score was assigned. i-mJOA scores are reported as mean ± standard deviation.

Analysis

Statistical analysis was carried out using SPSS V22 (Chicago, IL, USA). Bivariate analysis was conducted between variables using Pearson's and Spearman's correlations depending on the variable types involved. Wilcoxon signed-rank test was used to calculate significant changes in i-mJOA from primary care referral to surgical assessment. As cases were selected on the basis of having sufficient documentation, there was not missing data.

Results

Study demographics

1123 total scans were screened, resulting in a final cohort of 43 DCM cases (Figure 1). The vast majority of these scans were of unique patients, although rarely patients were re-imaged. The average age at time of symptom onset was 61.4±13.9 years and the majority of patients (28, 65%) were male. Of the 43 cases, 22 (51%) experienced previous myelopathy and the other 21 (49%) were presenting for the first time.

Heterogeneity in primary referrals of DCM patients points to the lack of a unified pathway

33 primary referrals were accessible to review. The majority of these were made

For peer review only

by a general practitioner (28, 85%) and less frequently by a physiotherapist (3, 9%) or other peripheral hospital (2, 6%). Half of all DCM cases were referred directly to a spine surgeon (22, 51%) whilst the other were seen by other specialties first (21, 49%), including neurology (N=16), orthopaedics (N=2), pain management (N=1), rheumatology (N=1) and geriatric medicine (N=1). Almost all cases (42, 98%) were ultimately reviewed by a spinal surgeon. Referral pathways are displayed separating new myelopathy cases from recurrent cases (Figure 2).

DCM patients encounter significant delays from the onset of symptoms to the time of primary referral

Symptom onset was defined as the point at which the patient first experienced symptoms. For example, if a primary referral stated the patient had been experiencing numbness in his fingers for 4 months. The onset of symptoms was defined as 4 months prior to the date of this referral. The times between symptom onset and primary referral for new and recurrent cases of DCM were 8.3±10.1 and 6.4±4.2 months respectively. 76% (16/21) of new cases and 24% (5/22) of recurrent cases of DCM were referred to an alternative specialist for secondary assessment rather than directly to a spine surgeon. The times between primary referral and secondary assessment for new and recurrent cases of DCM were 1.5±0.8 and 1.9±1.2 months respectively.

Secondary referral to spine surgery is consistently delayed and does not take patient characteristics into account

Following secondary assessment, all patients apart from one were then referred

For peer review only

on to see a spine surgeon. The one non-referred patient had a complex neurological and social background with mild myelopathy and was deemed better managed with regular neurology follow-up than consideration of spinal surgery. The times between secondary assessment and surgical assessment for new and recurrent cases of DCM were 5.1±4.0 and 5.5±2.2 months respectively. No clinical feature, patient demographic factor or specialty of secondary assessment was associated with a significantly different speed of referral to spinal surgeon review.

A minority of new DCM patients are directly referred to spine surgery

24% (5/21) of new cases and 77% (17/22) of recurrent cases were referred from primary assessment directly to a spine surgeon. The times between primary assessment and surgical assessment for these new and recurrent cases of DCM were 3.7±4.1 and 0.9±2.2 months respectively. The only factor associated with direct referral to a spinal surgeon was previous DCM ($p<0.001$). The overall times between symptom onset and surgical assessment for new and recurrent cases of DCM were 17.7±16.0 and 9.5±9.0 months respectively. Of the new cases of DCM, 45% (9/20) received an operation, 35% (7/20) received a follow-up appointment with a spine surgeon, and 20% (4/20) were discharged. Of the recurrent cases of DCM, 50% (11/22) received an operation, 23% (5/22) received a follow-up appointment with a spine surgeon, and 27% (6/22) were discharged.

Significant delays in MRI scans relative to symptom onset may contribute to delayed diagnosis of DCM

For peer review only - http://bmjopen.bmj.com/site/about/guidelines.xhtml

The observed cohort was formed on the basis of diagnostic cervical MRI scans, demonstrating spinal cord compression. Relative to symptom onset, the scan was performed 17.7±15.6 and 9.5±8.7 months after symptomatic onset for new and recurrent cases of DCM respectively. This scan was performed at 1.4±4.7 and 3.3±4.2 months after secondary assessment for new and recurrent cases of DCM respectively. Relative to surgical review it was performed at 2.3±0.7 months before and 0.5±1.2 months after surgical assessment for new and recurrent cases of DCM respectively. Thus, in general, imaging of new DCM cases was conducted prior to reaching a spinal surgeon, whereas recurrent cases were imaged after initial surgical consultation.

DCM patients continue to deteriorate along the pathway from referral to treatment

Between the earliest recorded assessment (primary or secondary) and surgical assessment, the following deteriorations in i-mJOA scores were observed (Figure 3). 59% of patients deteriorated, 24% remained stable, and 17% showed improvement, presumably due to symptom fluctuation or resolution of disc herniation. The total i-mJOA change for individual patients ranged from +4 to -6 points. On average, patients that deteriorated displayed a reduction of 2.3±1.8 i-mJOA points.

Discussion

The pathway from symptom onset to diagnosis and surgical assessment is complex and highly heterogeneous for DCM patients. They face long delays

For peer review only

before a diagnosis and further delays before surgical assessment. During this time, most experience a progression in symptoms. However, DCM symptoms are by at large not reversible. The observed deterioration is therefore likely to translate into increased long-term disability and lead to lower quality of life.

A diagnosis of DCM is made before reaching a spinal surgeon.

DCM patients principally enter the healthcare system via primary care. They are then referred via another specialty for assessment and imaging, typically Neurology, before reaching a spinal surgeon. This contrasts the study by Behrbalk et al, the only other study to consider the DCM health care system, which found most patients were initially assessed by orthopaedics.[18] A potential explanation is that patients may self-refer to Orthopedics in Israel but require a formal referral to Neurology or Neurosurgery. Regardless, both studies have demonstrated that the diagnosis of new onset DCM is made outside of spinal surgery.

This finding is significant as DCM treatment and research is largely led by spinal surgeons. In order to accelerate diagnosis and improve outcomes based on existing treatment paradigms, greater clarity of the interaction between other specialties and DCM patients is critical.

DCM patients wait months for a diagnosis and years for treatment

Delays in patient pathways were noted in all components of the system. The greatest delay appears to exist between disease onset and primary referral to

For peer review only

secondary care. This is consistent with Behbalk et al and patient experience.[18]

The cause for this delay is likely to be multi-factorial. Potential factors include late presentation by patients, subtle or non-specific symptoms and examination findings,[24,25] misdiagnoses and a lack of awareness of DCM. Future studies are required to determine the exact reasons for pre-referral delays in order to develop targeted interventions.

For peer review only

Minimising delays of treatment is especially important in DCM, because length of symptoms and disease severity are the two most important factors that determine response to surgery of DCM patients [13-15]. Moreover, shortening the times until patients are seen by a myelopathy specialist is likely to be the single most effective strategy of improving patient outcomes.

The lack of a unified pathway is likely to contribute to delays in treatment

The majority of new cases (76%) were not directly referred to a spine surgeon but instead were reviewed by neurologists, orthopaedics, pain specialists, rheumatologists, and geriatricians. The specialty performing the secondary assessment did not influence referral speed from secondary care to spinal surgery. These findings corroborate that there is no streamlined pathway for surgical assessment of a patient with DCM.

At the early stages, symptoms are often subtle and often missed by non-specialists. However, even asymptomatic patients with MRI-proven cervical spinal cord compression run a considerable risk of developing myelopathy. A

recent study found that 59% of asymptomatic individuals over 40 had cervical cord compression on MRI.[26] A further study observed that 13% of non-myelopathic individuals with cervical cord compression developed myelopathy within a 2-year period.[27]

Our data indicated that disease severity did not influence speed of referral to a spine surgeon. This may seem counter-intuitive but potentially be explained by the broad range of symptoms associated with myelopathy.

For these reasons, the authors advocate more rapid assessment by a specialist service once a patient has proven cord compression, especially alongside clinically myelopathic features. This is also reflected in the first recent international guidelines.[10]

DCM patients continue to deteriorate whilst they wait for treatment

This study found that 59% of patients deteriorated between their earliest available assessment and the point at which they reached assessment by a spine surgeon. The minimum clinically important difference (MCID) in DCM varies by disease severity:[28,29] 1 point for mild disease (mJOA ≥ 15), 2 points for moderate disease (mJOA score of 12–14), and 3 points for severe disease (mJOA score < 12). In the deteriorating group, all but one patient were found to have mild disease (mJOA ≥ 15) at initial assessment. Within this group, the average deterioration was 2.3 i-mJOA points, demonstrating clinically significant disease progression. More rapid DCM referral services and streamlined pathways to specialist assessment could help to prevent this source of potentially non-

For peer review only

reversible deterioration.

What would the ideal pathway be for assessment and management of DCM?

As it stands, this data shows new diagnosis and management as a two-stage process. Stage one: patients receive a diagnosis of DCM outside of spinal surgery based upon clinical symptomatology and MRI findings. Stage two: onward referral to spinal surgery and surgery is offered if indicated.

Whilst direct referral to spinal surgery upon initial clinical suspicion would significantly shorten this process, it would likely lead to a number of problems. Firstly, given the nature of DCM's non-specific symptoms, especially early on, and the limited access in the UK to MRI imaging within primary care, this would lead to many inappropriate referrals and place a strain on already stressed outpatient spinal services. Secondly, these inappropriate referrals are then being assessed by specialists, who can confidently consider DCM, but will not be best placed or experienced to consider other differentials. Therefore, the exclusion of myelopathy could place delays on other diagnoses.

Similar dilemmas have been addressed for many other conditions using intermediate pathways. For example, in the UK, we have a 'musculoskeletal' pathway triaging complaints such as lower back pain and joint pain. In this pathway, patients are referred to physiotherapy led assessment centers, with access to investigations such as MR imaging and electrophysiology. These centers have access to specialist MDTs, who help to decide whether patients can be managed locally or referred onto specialist services. A recent systematic

For peer review only

review has shown such triage was concordant with specialists 68 – 96% of the time and reduced referrals to specialist clinics by 20 – 60%.[30]

At present, the 'musculoskeletal' service is better geared to classically 'orthopaedic' or 'rheumatological' conditions and a potential weakness for DCM would be neurological mimics. In this study, only 9% of referrals came from this musculoskeletal service, with most patients being referred onto Neurology. A more refined assessment process will require further research. Building on the existing intermediate pathway with closer involvement of neurology might be a sensible approach.

For recurrent disease, this study shows that direct access to spinal surgery is appropriate. Of the patients referred, 50% received operations and 23% were kept under surveillance. We speculate that these patients' previous DCM diagnosis heightened awareness by patient and healthcare practitioner, leading to a greater confidence in direct referral to spinal services.

Limitations

Whilst this study was conducted in a single neurosciences centre, it serves a large regional population, almost a tenth of the UK population as a whole.[31] Additionally, given the UK has a National Health Service that delivers care within common structures, the findings in this study are likely generalizable throughout at least the UK health system.

For peer review only

The epidemiology of DCM is poorly characterized, however it is thought to be a common condition.[32] The identification of only 43 cases of DCM over 1 year was lower than expected. However, the majority of patients receiving treatment at this neurosciences centre present with external imaging obtained in referring secondary centres, which did not facilitate access to full clinical records as described in the methodology. Hence, these patients were not included in the present cohort. The low number therefore is likely to reflect the role of the neuroradiology department acting as a secondary centre serving a more local population.

The retrospective nature of the study lead to the development of i-mJOA. While true mJOA would have been preferable, the strong kappa statistics and low average difference indicate that total i-mJOA does represent a reliable proxy and broad-brush method of demonstrating disease severity and changes in disease severity over time retrospectively.

Conclusions

This work demonstrates that patients experience significant delays in diagnosis and time to surgical review. During this time, disease progression occurred and patients functionally deteriorated. The best post-operative outcomes for DCM patients are attained with rapid surgical review and intervention when appropriate. Our findings call for the formation of dedicated streamlined pathways to surgical review for patients with spinal cord compression on MRI, especially in the context of clinical myelopathy. Further prospective system

For peer review only

characterization of factors associated with patient flow is needed to introduce specific targeted interventions to improve the management of DCM in the UK.

Table 1. i-mJOA methodology.

Figure 1. Flow diagram depicting cohort formation methodology.

Figure 2. Patient referral pathways from symptom onset for recurrent cases of DCM (left) and new cases of DCM (right). Times are given in the format: average months ± standard deviation (number of cases). Dashed lines represent total time durations, not a specific patient pathway.

Figure 3. i-mJOA scores at each clinical phase of assessment. Mean ± standard deviation.

References

1. Nouri A, Tetreault L, Singh A, Karadimas SK, Fehlings MG. Degenerative Cervical Myelopathy: Epidemiology, Genetics, and Pathogenesis. Spine (Phila Pa 1976). 2015 Jun 15;40(12):E675-93

2. Bednarik J, Kadanka Z, Dusek L., et al. Presymptomatic spondylotic cervical myelopathy: an updated predictive model. Eur Spine J. 2008 Mar; 17(3): 421–431.

3. King JTJ, McGinnis KA, Roberts MS. Quality of Life Assessment with the Medical Outcomes Study Short Form-36 among Patients with Cervical Spondylotic Myelopathy. Neurosurgery. 2003 Jan 1;52(1):113.

4. de Oliveira Vilaça C, Orsini M, Leite MA, de Freitas MR, Davidovich E, Fiorelli R, Fiorelli S, Fiorelli C, Oliveira AB, Pessoa BL. Cervical Spondylotic Myelopathy: What the Neurologist Should Know. Neurol Int. 2016 Nov 23;8(4):6330

5. Matz PG, Anderson PA, Holly LT, Groff MW, Heary RF, Kaiser MG, et al.: The natural history of cervical spondylotic myelopathy. J Neurosurg Spine. 2009 Aug;11(2):104-11.

For peer review only

6. Davies, B. M., McHugh, M., Elgheriani, A., Kolias, A. G., Tetreault, L., Hutchinson, P. J. A., et al. The reporting of study and population characteristics in degenerative cervical myelopathy: A systematic review. PloS One, 12(3), e0172564.

7. Fehlings MG, Wilson JR, Kopjar B, et al. Efficacy and safety of surgical decompression in patients with cervical spondylotic myelopathy: results of the AOSpine North America prospective multi-center study. J Bone Joint Surg Am 2013;95:1651–8.

8. Fehlings MG, Ibrahim A, Tetreault L, et al. A global perspective on the outcomes of surgical decompression in patients with cervical spondylotic myelopathy: results from the prospective multicenter AOSpine international study on 479 patients. Spine (Phila Pa 1976). 2015 Sep 1;40(17):1322-8.

9. Oh, T., Lafage, R., Lafage, V., et al. Comparing Quality of Life in Cervical Spondylotic Myelopathy with Other Chronic Debilitating Diseases Using the SF-36 Survey. World Neurosurg. 2017 Oct;106:699-706.

10. Fehlings MG, Tetreault L, Riew K, et al. A Clinical Practice Guideline for the Management of Patients With Degenerative Cervical Myelopathy: Recommendations for Patients With Mild, Moderate, and Severe Disease and Nonmyelopathic Patients With Evidence of Cord Compression. Global Spine Journal. 2017 Sept. 7(3_suppl), 70S–83S.

11. Matz PG, Anderson PA, Groff MW, et al. Cervical laminoplasty for the treatment of cervical degenerative myelopathy. J Neurosurg Spine 11:157–169, 2009

12. Ebersold MJ, Pare MC, Quast LM. Surgical treatment for cervical spondylitic myelopathy. J Neurosurg. 1995 May;82(5):745-51.

13. Holly LT, Matz PG, Anderson PA, et al. Clinical prognostic indicators of surgical outcome in cervical spondylotic myelopathy. J Neurosurg Spine 11:112–118, 2009

14. Zhang JT, Wang LF, Wang S, Li J, Shen Y. Risk factors for poor outcome of surgery for cervical spondylotic myelopathy. Spinal Cord. 2016 Dec;54(12):1127-1131

15. Tetreault L, Kopjar B, Côté P, Arnold P, Fehlings MG. A Clinical Prediction Rule for Functional Outcomes in Patients Undergoing Surgery for Degenerative Cervical Myelopathy: Analysis of an International Prospective Multicenter Data Set of 757 Subjects. J Bone Joint Surg Am. 2015 Dec 16;97(24):2038-46

16. Nouri A, Tetreault L, Côté P, Zamorano JJ, Dalzell K, Fehlings MG. Does Magnetic Resonance Imaging Improve the Predictive Performance of a Validated Clinical Prediction Rule Developed to Evaluate Surgical Outcome in Patients With Degenerative Cervical Myelopathy? Spine (Phila Pa 1976). 2015 Jul

For peer review only

15;40(14):1092-100.

17. "Elemental Ideas - Cervical Spondylotic Myelopathy." Cambridge TV. www.myelopathy.org, www.cambridge-tv.co.uk/tag/myelopathy/.

18. Behrbalk E, Salame K, Regev GJ, Keynan O, Boszczyk B, Lidar Z. Delayed diagnosis of cervical spondylotic myelopathy by primary care physicians. Neurosurg Focus. 2013 Jul;35(1):E1

19. "Routes to Diagnosis." Routes to Diagnosis. Macmillan Cancer Support, n.d. eb. 21 May 2017.

20. Buvarp Dyrop H, Vedsted P, Rædkjær M, Safwat A, Keller J. Routes to Diagnosis for Suspected Sarcoma: The Impact of Symptoms and Clinical Findings on the Diagnostic Process. Sarcoma. 2016;2016:8639272. doi: 10.1155/2016/8639272.

21. Walter FM et al. Symptoms and patient factors associated with diagnostic intervals for pancreatic cancer (SYMPTOM pancreatic study): a prospective cohort study. Lancet Gastroenterol Hepatol. 2016 Dec;1(4):298-306.

22. Walter FM et al. Symptoms and patient factors associated with longer time to diagnosis for colorectal cancer: results from a prospective cohort study. Br J Cancer. 2016 Aug 23;115(5):533-41.

23. Davies BM, McHugh M, Elgheriani A, et al. Reported Outcome Measures in Degenerative Cervical Myelopathy: A Systematic Review. PLoS One. 2016 Aug 2;11(8)

24. Rhee JM, Heflin JA, Hamasaki T, Freedman B. Prevalence of physical signs in cervical myelopathy: a prospective, controlled study. Spine (Phila Pa 1976). 2009 Apr 20;34(9):890-5

25. Cook CE, Wilhelm M, Cook AE, Petrosino C, Isaacs R. Clinical tests for screening and diagnosis of cervical spine myelopathy: a systematic review. J Manipulative Physiol Ther. 2011 Oct;34(8):539-46.

26. Kovalova I, Kerkovsky M, Kadanka Z, et al. Prevalence and Imaging Characteristics of Nonmyelopathic and Myelopathic Spondylotic Cervical Cord Compression. Spine (Phila Pa 1976). 2016 Dec 15;41(24):1908-1916.

27. Zdenek Kadanka, Jr, Blanka Adamova, Milos Kerkovsky, et al. Predictors of symptomatic myelopathy in degenerative cervical spinal cord compression. Brain Behav. 2017 Sep; 7(9): e00797.

28. Tetreault L, Nouri A, Kopjar B, Côté P, Fehlings MG: The minimum clinically important difference of the Modified Japanese Orthopaedic Association Scale in patients with degenerative cervical myelopathy. Spine (Phila Pa 1976) 40:1653–1659, 2015

For peer review only

29. Tetreault L, Wilson JR, Kotter MR, et al. Predicting the minimum clinically important difference in patients undergoing surgery for the treatment of degenerative cervical myelopathy. Neurosurg Focus. 2016 Jun;40(6):E14

30. Hussenbux, A., Morrissey, D., Joseph, C., & McClellan, C. M. Intermediate care pathways for musculoskeletal conditions--are they working? A systematic review. Physiotherapy. 2015 Mar;101(1):13-24.

31. East of England Trauma Network, NHS, www.eoetraumanetwork.nhs.uk/. October 2016.

32. Davies BM, Mowforth OD, Smith EK, Kotter MR. Degenerative cervical myelopathy. BMJ. 2018 Feb 22;360:k186

For peer review only

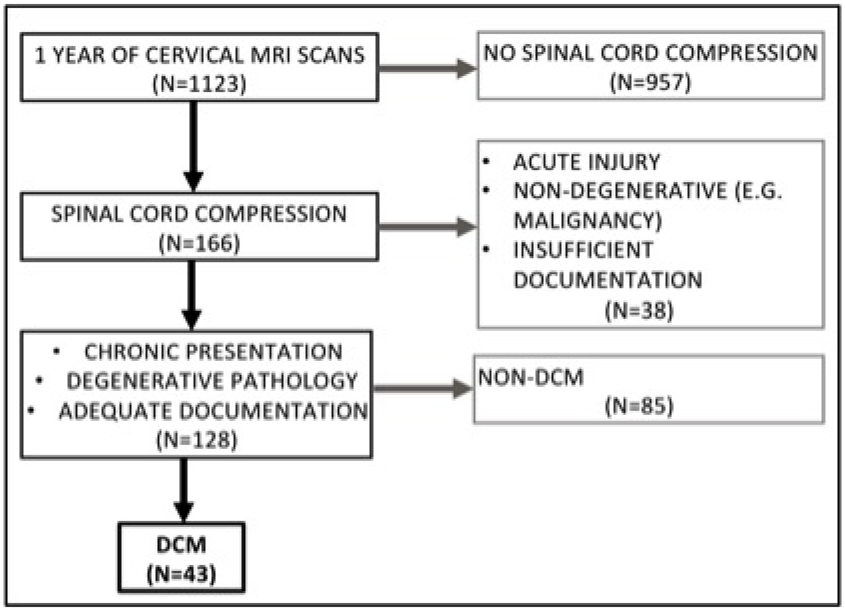

Figure 1. PRISMA flow diagram depicting cohort formation methodology.

35x25mm (300 x 300 DPI)

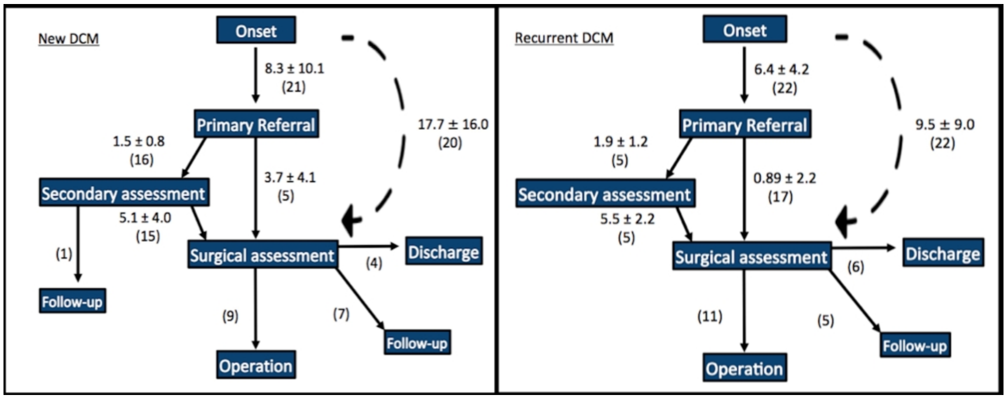

Figure 2. Patient referral pathways from symptom onset for recurrent cases of DCM (left) and new cases of DCM (right). Times are given in the format: average months ± standard deviation (number of cases). Dashed lines represent total time durations, not a specific patient pathway.

426x166mm (300 x 300 DPI)

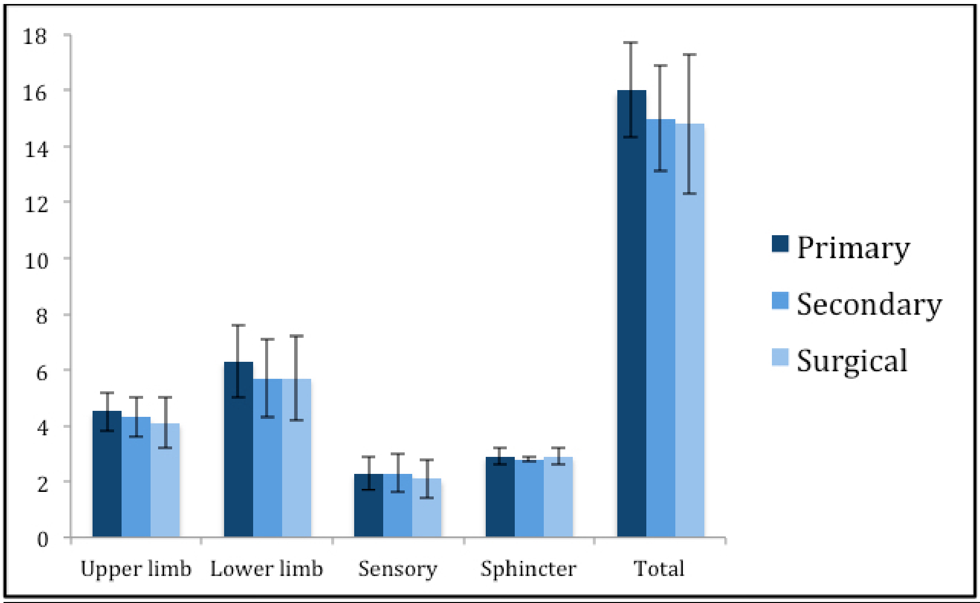

	Primary	Secondary	Surgical
**Upper limb**	4.5±0.7	4.3±0.7	4.1±0.90
**Lower limb**	6.3±1.3	5.7±1.4	5.7±1.5
**Sensory**	2.3±0.6	2.3±0.7	2.1±0.7
**Sphincter**	2.9±0.3	2.8±0.1	2.9±0.3
**Total**	16.0±1.7	15.0±1.9	14.8±2.5

Figure 3. i-mJOA scores at each clinical phase of assessment. Mean ± standard deviation.

55x45mm (300 x 300 DPI)

STROBE Statement

Checklist of items that should be included in reports of observational studies

Section/Topic	Item No	Recommendation	Reported on Page No
**Title and abstract**	1	(*a*) Indicate the study's design with a commonly used term in the title or the abstract	1
		(*b*) Provide in the abstract an informative and balanced summary of what was done and what was found	3
**Introduction**			
Background/rationale	2	Explain the scientific background and rationale for the investigation being reported	5
Objectives	3	State specific objectives, including any prespecified hypotheses	7
**Methods**			
Study design	4	Present key elements of study design early in the paper	7
Setting	5	Describe the setting, locations, and relevant dates, including periods of recruitment, exposure, follow-up, and data collection	7
Participants	6	(*a*) *Cohort study*—Give the eligibility criteria, and the sources and methods of selection of participants. Describe methods of follow-up<br>*Case-control study*—Give the eligibility criteria, and the sources and methods of case ascertainment and control selection. Give the rationale for the choice of cases and controls<br>*Cross-sectional study*—Give the eligibility criteria, and the sources and methods of selection of participants	7
		(*b*) *Cohort study*—For matched studies, give matching criteria and number of exposed and unexposed<br>*Case-control study*—For matched studies, give matching criteria and the number of controls per case	
Variables	7	Clearly define all outcomes, exposures, predictors, potential confounders, and effect modifiers. Give diagnostic criteria, if applicable	8,9
Data sources/measurement	8*	For each variable of interest, give sources of data and details of methods of assessment (measurement). Describe comparability of assessment methods if there is more than one group	8,9
Bias	9	Describe any efforts to address potential sources of bias	10
Study size	10	Explain how the study size was arrived at	11
Quantitative variables	11	Explain how quantitative variables were handled in the analyses. If applicable, describe which groupings were chosen and why	10
Statistical methods	12	(*a*) Describe all statistical methods, including those used to control for confounding	10
		(*b*) Describe any methods used to examine subgroups and interactions	10
		(*c*) Explain how missing data were addressed	10
		(*d*) *Cohort study*—If applicable, explain how loss to follow-up was addressed<br>*Case-control study*—If applicable, explain how matching of cases and controls was addressed<br>*Cross-sectional study*—If applicable, describe analytical methods taking account of sampling strategy	10
		(*e*) Describe any sensitivity analyses	10

For peer review only - http://bmjopen.bmj.com/site/about/guidelines.xhtml

Section/Topic	Item No	Recommendation	Reported on Page No
**Results**			
Participants	13*	(a) Report numbers of individuals at each stage of study—eg numbers potentially eligible, examined for eligibility, confirmed eligible, included in the study, completing follow-up, and analysed	7
		(b) Give reasons for non-participation at each stage	
		(c) Consider use of a flow diagram	
Descriptive data	14*	(a) Give characteristics of study participants (eg demographic, clinical, social) and information on exposures and potential confounders	11
		(b) Indicate number of participants with missing data for each variable of interest	
		(c) *Cohort study*—Summarise follow-up time (eg, average and total amount)	
Outcome data	15*	*Cohort study*—Report numbers of outcome events or summary measures over time	
		Case-control study—Report numbers in each exposure category, or summary measures of exposure	
		Cross-sectional study—Report numbers of outcome events or summary measures	11
Main results	16	(*a*) Give unadjusted estimates and, if applicable, confounder-adjusted estimates and their precision (eg, 95% confidence interval). Make clear which confounders were adjusted for and why they were included	11-18
		(*b*) Report category boundaries when continuous variables were categorized	
		(*c*) If relevant, consider translating estimates of relative risk into absolute risk for a meaningful time period	
Other analyses	17	Report other analyses done—eg analyses of subgroups and interactions, and sensitivity analyses	9
**Discussion**			
Key results	18	Summarise key results with reference to study objectives	20
Limitations	19	Discuss limitations of the study, taking into account sources of potential bias or imprecision. Discuss both direction and magnitude of any potential bias	19
Interpretation	20	Give a cautious overall interpretation of results considering objectives, limitations, multiplicity of analyses, results from similar studies, and other relevant evidence	20
Generalisability	21	Discuss the generalisability (external validity) of the study results	19
**Other Information**			
Funding	22	Give the source of funding and the role of the funders for the present study and, if applicable, for the original study on which the present article is based	1

**Give information separately for cases and controls in case-control studies and, if applicable, for exposed and unexposed groups in cohort and cross-sectional studies.*

Note: An Explanation and Elaboration article discusses each checklist item and gives methodological background and published examples of transparent reporting. The STROBE checklist is best used in conjunction with this article (freely available on the Web sites of PLoS Medicine at http://www.plosmedicine.org/, Annals of Internal Medicine at http://www.annals.org/, and Epidemiology at http://www.epidem.com/). Information on the STROBE Initiative is available at www.strobe-statement.org.

For peer review only - http://bmjopen.bmj.com/site/about/guidelines.xhtml
